# Chloride Gradient Is Involved in Ammonium Influx in Human Erythrocytes

**DOI:** 10.3390/ijms25137390

**Published:** 2024-07-05

**Authors:** Julia Sudnitsyna, Tamara O. Ruzhnikova, Mikhail A. Panteleev, Alexandra Kharazova, Stepan Gambaryan, Igor V. Mindukshev

**Affiliations:** 1 Center for Theoretical Problems of Physicochemical Pharmacology, Russian Academy of Sciences, 30 Srednyaya Kalitnikovskaya St., 109029 Moscow, Russia; mapanteleev@yandex.ru; 2 Sechenov Institute of Evolutionary Physiology and Biochemistry, Russian Academy of Sciences, 44 Thorez Ave., 194223 Saint Petersburg, Russia; ruzhnikova.tamara@gmail.com (T.O.R.); iv_mindukshev@mail.ru (I.V.M.); 3 Department of Cytology and Histology, Saint Petersburg State University, 7/9 Universitetskaya Emb., 199034 Saint Petersburg, Russia; akharazova@gmail.com

**Keywords:** erythrocytes, anion exchanger 1, band 3, DIDS, DIBAC_4_(3), RhAG, ammonia, ammonium, chloride gradient

## Abstract

The ammonia/ammonium (NH_3_/NH_4_^+^, AM) concentration in human erythrocytes (RBCs) is significantly higher than in plasma. Two main possible mechanisms for AM transport, including simple and facilitated diffusion, are described; however, the driving force for AM transport is not yet fully characterized. Since the erythroid ammonium channel RhAG forms a structural unit with anion exchanger 1 (eAE1) within the ankyrin core complex, we hypothesized the involvement of eAE1 in AM transport. To evaluate the functional interaction between eAE1 and RhAG, we used a unique feature of RBCs to swell and lyse in isotonic NH_4_^+^ buffer. The kinetics of cell swelling and lysis were analyzed by flow cytometry and an original laser diffraction method, adapted for accurate volume sensing. The eAE1 role was revealed according to (i) the changes in cell swelling and lysis kinetics, and (ii) changes in intracellular pH, triggered by eAE1 inhibition or the modulation of eAE1 main ligand concentrations (Cl^−^ and HCO_3_^−^). Additionally, the AM import kinetics was analyzed enzymatically and colorimetrically. In NH_4_^+^ buffer, RBCs concentration-dependently swelled and lysed when [NH_4_^+^] exceeded 100 mM. Cell swelling and hemolysis were tightly regulated by chloride concentration. The complete substitution of chloride with glutamate prevented NH_4_^+^-induced cell swelling and hemolysis, and the restoration of [Cl^−^] dose-dependently amplified the rates of RBC swelling and lysis and the percentage of hemolyzed cells. Similarly, eAE1 inhibition impeded cell swelling and completely prevented hemolysis. Accordingly, eAE1 inhibition, or a lack of chloride anions in the buffer, significantly decreased NH_4_^+^ import. Our data indicate that the eAE1-mediated chloride gradient is required for AM transport. Taken together, our data reveal a new player in AM transport in RBCs.

## 1. Introduction

Na^+^-independent anion exchanger 1 (AE1) mediates the electroneutral transmembrane chloride-bicarbonate (Cl^−^/HCO_3_^−^) exchange thus contributing to the maintenance of intracellular pH (pH_i_), cell volume, acid-base balance, and membrane potential via its contribution to the control of the transmembrane Cl^−^ gradient. AE1, also referred to as the band 3 protein, is expressed in the erythroid membrane (eAE1) and as an N-terminally truncated variant (kAE1) in the α-intercalated (α-IC) cells of the kidney collecting duct [1,2]. In red blood cells (RBCs), the eAE1 isoform (10^6^ copies per cell), in addition to anion transport, has several other vital features including a crucial role in membrane integrity maintenance [3,4,5,6,7,8,9]. eAE1 is central for the proteins of the eAE1 tetrameric complex (eAE1, glycophorin A (GPA), protein 4.2, carbonic anhydrase II (CAII)) interacting with the Rhesus subcomplex (RhAG, RhCE, RhD, CD47, ICAM-4, glycophorin B (GPB)) via the direct association of eAE1, protein 4.2, and RhAG with ankyrin [8,9,10,11]. In eAE1 knockouts (KOs), a secondary erythrocyte membrane protein deficit was detected, including, among others, a severe reduction in the Rh subcomplex proteins [12]. These data indicated that eAE1 possibly forms the core of a larger sub-macrocomplex [6,13,14,15,16] and Rh complex proteins and eAE1 could associate either directly or indirectly through their common interaction with ankyrin-R [9,17]. However, it has not been fully elucidated yet what the physiological relevance of eAE1 colocalization is and its interaction with the RhAG channel [11,16,18,19].

In humans, ammonium/ammonia (AM) transport glycoproteins of the Rhesus family (RhBG and RhCG) are expressed in tissues involved in AM generation, secretion, and excretion, such as kidney, liver, etc., [17,20,21,22]. RhCG, RhBG, and their erythroid ortholog RhAG expression in various heterologous cell models facilitate the influx of ammonia/ammonium [23]. RhBG and RhCG have been extensively studied over the last decade, mainly in the kidneys [11,21,23,24,25]. Both RhBG and RhCG were found to contribute to the uptake of ammonia in the basolateral membrane in mice [24]. In RhBG-transfected Madin–Darby canine kidney cells, it was found to be attached to the membrane skeleton through its interaction with ankyrin-G, similar to the erythroid Rh subcomplex with ankyrin-R [16], and this linkage was essential for NH_3_ transport [26]. As well as in erythrocytes, the renal ammonium channel RhBG and the renal AE1 exchanger have been shown by coimmunoprecipitation to colocalize in the basolateral domain of α-IC cells. Further analysis of AM fluxes according to alkalinization rates in NH_4_^+^ gradients confirmed the functional cooperation of these proteins to facilitate ammonium and proton excretion in urine [11,16,24,27]. In the kidneys, the physiological relevance of RhBG and RhCG is at least partly clear; however, the role of the erythrocyte-restricted ammonium channel RhAG expression remains unclear [17,18,28]. 

At the same time, the question whether erythrocytes are able to trap/transport/release AM at all is still a question for debate. However, here are the facts strongly suggesting this feature of erythrocytes. First, RBCs are the only blood cells that swell and lyse in isotonic media containing NH_4_^+^ [29,30,31], indicating the presence of an AM-transporting system. Secondly, the intracellular AM concentration in RBCs was found to be three to seven times higher [32,33,34,35] than that of plasma (10–60 µM) [32,36,37,38]. Beyond that, it was shown that in humans with altered or depleted RhAG expression, or RhAG KO mice, RBCs exhibited a reduction in AM transport that strictly correlated with the level of their RhAG expression [39,40,41]. 

Assuming the possibility of AM transport in RBCs, AM influx in RBCs should trigger the following events: (a) pHi increase [4,39], (b) osmolality increase caused by the coupling chloride influx to compensate for the increased pHi (net NH_4_Cl influx), (c) AQP1-mediated water influx to compensate the increased osmolality [42,43], and (d) corresponding volume increase. Previously, two main possible protein-facilitated mechanisms for Rhesus glycoprotein-mediated transport were described, including NH_3_ uniport and NH_4_^+^/H^+^ antiport [44,45,46]. If RBCs are capable of trapping AM (either protonated or neutral), the influx should theoretically occur against the gradient which requires the energy. However, the question of either mechanism, AM influx in RBCs, or the driving force for it, is still under debate, partly due to the absence of a specific inhibitor for Rhesus glycoproteins.

Based on previously published data on the structural coupling of RhAG and eAE1 [18], our preliminary findings on the possible functional interaction of RhAG and eAE1 [29], and data on the functional coupling of kAE1 and RhBG in the kidneys [11,25], we hypothesized that eAE1 plays a significant role in the AM transport mechanism in human RBCs. 

In this study, using enzymatic and colorimetric approaches, we confirmed and evaluated the capacity of RBCs to transport AM. Based on the RBC unique feature to swell and lyse in isosmotic NH_4_^+^ buffer, we developed an original method for the indirect assessment and quantitative characterization of AM transport kinetics in RBCs. To reveal the eAE1 role in AM transport, we inhibited eAE1 and modulated eAE1 ligand concentration, showing that these interventions significantly affected AM transport and the corresponding AM influx. Our data clearly demonstrated that eAE1 is involved in AM transport, and that the eAE1-mediated chloride gradient is crucial for AM transport in RBCs.

## 2. Results

### 2.1. Swelling and Hemolysis in Isotonic NH_4_^+^ Buffer Is the Unique Feature of Human RBC

Ammonia is a weak base with a pKa of 8.95 at 37 °C [47]. At physiological conditions (pH 7.4, 37 °C), according to the Henderson–Hasselbach equation, almost 98% of AM remains in the charged form of NH_4_^+^ and only up to 2% in the uncharged form of NH_3_ [32,48]. Therefore, NH_3_ simple diffusion cannot solely be a cause of AM-induced hemolysis. Thus, some transporting systems for the charged species should be present in RBCs. In a series of studies that evaluated AM transport according to AM-triggered pHi changes, the NH_4_Cl concentrations used did not exceed 30 mM [4,40,45,49]. However, to cause fatal swelling of RBCs and to lead to complete hemolysis (e.g., for leukocyte isolation or in hematological analyzers), a much higher NH_4_Cl concentration (140 mM) is required. Therefore, to study the AM-triggered RBC swelling and lysis, we created a model of an ammonium stress test (AST) with complete substitution of sodium chloride with ammonium chloride in HEPES buffer (NH_4_^+^ buffer; see Section 4.1).

**Figure 1:**
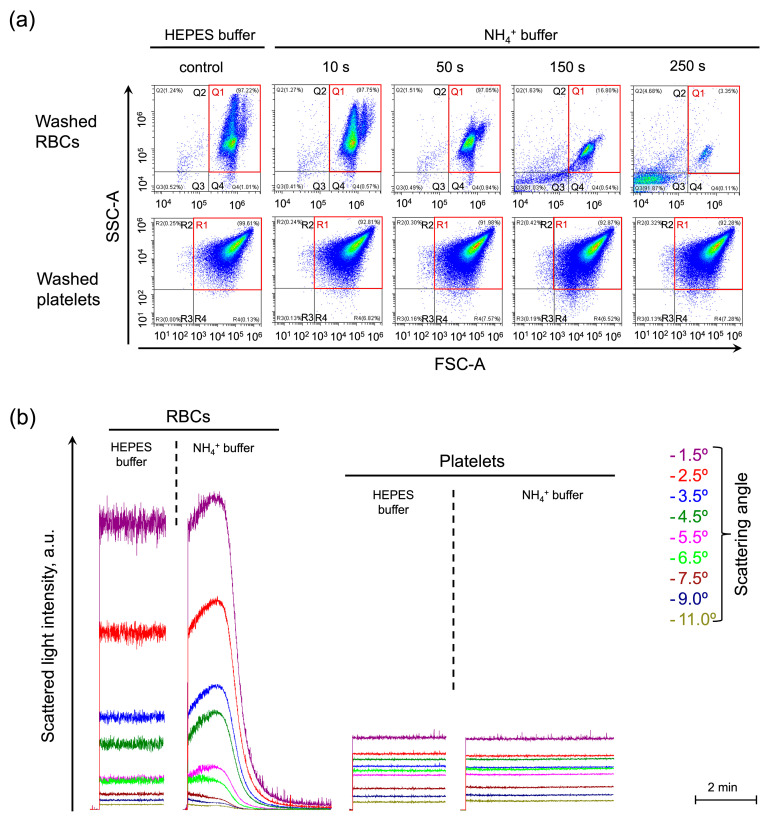
Swelling and hemolysis in NH_4_^+^ buffer is the unique reaction of RBCs among the blood cells. (**a**) Washed RBCs (10^6^ cells/mL) or platelets (10^6^ cells/mL) were suspended in HEPES buffer for control and analyzed by flow cytometry. Next, the cells were suspended in an NH_4_^+^ buffer and immediately analyzed in a TIME mode with SSC-A/FSC-A plots visualization at selected time points as indicated. The control gates, Q1 for RBCs and R1 for platelets, are framed in red. Gates Q2 and Q3 (R2 and R3 for platelets) characterize the hemolyzed cells. Gate Q4 (R4 for platelets) characterizes cells with decreased granularity; The x–y scaling is preserved in all scatter-plots. (**b**) The cells were prepared and processed as described in (**a**) and analyzed using the laser diffraction method at 37 °C. SLI from RBCs suspended in the HEPES buffer was recorded at 1.5, 2.5, 3.5, 5.5, 6.5, 7.5, 9, and 11° angles for 2 min for control. Then RBCs (10^6^ cells/mL) were suspended in isotonic NH_4_^+^ buffer and SLI registration continued for 4 min, the increase in SLI in the range of angles from 1 to 6° corresponded to cell swelling, the SLI decrease throughout the scattering angles to hemolysis. Next, the washed platelets (10^6^ cells/mL) were suspended in HEPES buffer and then in isotonic NH_4_^+^ buffer with the corresponding SLI registration.

The widely used Coulter method of cell volume estimation was not applicable for use in the AST model because it is also based on the osmotic properties of cells [50]. Therefore, first, using the light scattering-based approaches including single-cell analysis by flow cytometry and laser diffraction analysis of the cells in an ensemble, we showed in kinetics, that other cells that lack an ammonium channel, e.g., platelets, do not swell and lyse in isotonic NH_4_^+^ buffer (Figure 1a,b). In washed platelets, no significant differences in either event count, or the distribution in frontside scattering (FSC)/side scattering (SSC) coordinates were detected during the AST from control (Figure 1a, washed platelets, gate R). The RBC’s distribution on the scatter plot was not monomodal, but bimodal (two “populations” of RBCs, Figure 1a, washed RBCs, gate Q1), due to the effect of the erythroid non-spheric shape on forward scattering distribution. The RBCs were characterized by a decrease in cell volume distribution width (Figure 1a, washed RBCs, red gate Q1, 10 and 50 s) followed by massive hemolysis with only up to 3% of cells remaining in the control gate Q1 by the time-point of 250 s (Figure 1a, washed RBCs, red gate Q1, 50–250 s). Using laser diffraction, we also showed that, during AST, RBCs rapidly swelled and underwent lysis within 2 min, whereas platelets did not show any significant change in SLI during the AST (Figure 1b). 

Therefore, here, using flow cytometry (Figure 1a) and laser diffraction (Figure 1b), we showed that swelling and hemolysis in isotonic NH_4_^+^ buffer is a reaction unique for red blood cells. 

### 2.2. AM-Induced RBC Swelling and Hemolysis Dose-Dependently Correlate with [NH_4_^+^] in the Buffer

**Figure 2:**
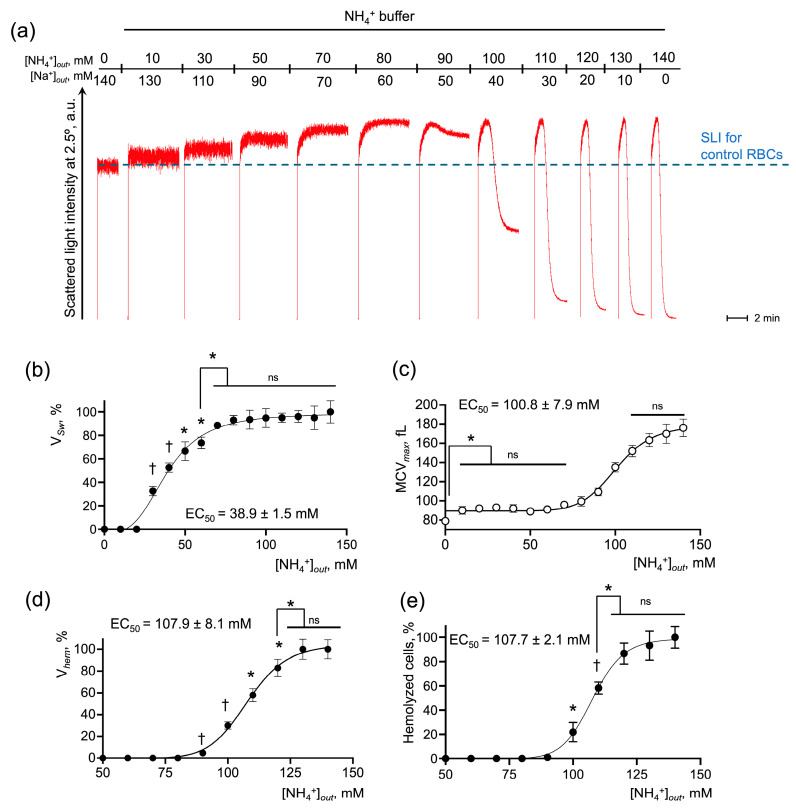
RBC swelling and hemolysis directly correlate with NH_4_^+^ concentration in the buffer. (**a**) Washed RBCs (10^6^ cells/mL) were suspended in HEPES buffer and SLI at 2.5° angle was registered for 2 min for the control. Then the RBCs (10^6^ cells/mL) were sequentially suspended in isotonic NH_4_^+^ buffers with the indicated concentrations of NH_4_^+^ and Na^+^ followed by the SLI registration for 3–4 min for each AM concentration. Representative experiment, one of 6; data from (**a**) and its replications were analyzed and the values for V*_Sw_* (**b**), maximum mean cell volume—MCV*_max_* (**c**), V*_hem_* (**d**), and % hemolysis (**e**) were plotted against NH_4_^+^ concentration for the following calculation of half-maximal effective ligand concentration (EC_50_). Data are presented as Mean ± SD (n = 6). One-way ANOVA, Leven’s test *p* > 0.05, Tukey HSD post hoc, *, *p* < 0.05, †, *p* < 0.01, ns—not significant.

Next, we analyzed the AM-triggered RBC swelling and hemolysis and established the parameters for its quantitative characterization (Supplementary Figures S1–S3). AM concentration-dependently increased RBC swelling and hemolysis degrees in NH_4_^+^ buffer (Figure 2a). The rate of cell swelling (V*_Sw_*), rate and percent of hemolysis (V*_hem_* and %Hem, correspondingly), and maximal cell volume reached during AST (MCV*_max_*), were determined for each experiment and plotted against the NH_4_^+^ concentration to obtain dose-response curves (Figure 2b–e). Analysis of the dose dependencies revealed that V*_Sw_*, V*_hem_*, % hemolysis, and MCV*_max_* were characterized by saturation and therefore the Hill model was appropriate for their description. Next, the relative half-effective concentrations (EC_50_) for each parameter were calculated (Figure 2a,c,d). Additionally, the maximal increase in MCV was analyzed for each AM concentration (Figure 2b). V*_Sw_* was undetectable until AM concentration in the NH_4_^+^ buffer increased to 50 mM (Figure 2b). Once AM concentration in the NH_4_^+^ buffer reached 70 mM, V*_Sw_* reached the plateau with the maximum rate of cell swelling (Figure 2b). MCV*_max_* was characterized by two plateaus: (i) [NH_4_^+^] from 10 to 70 mM caused a slight and significant increase in MCV from 85 fL ± 3 fL to 95 fL ± 2 fL; and (ii) [NH_4_^+^] from 125 to 140 mM caused an increase in MCV*_max_* up to 165 fL ± 8 fL (Figure 2c). The hemolysis rate V*_hem_* and the percent of hemolyzed cells exhibited dose dependencies with similar characteristics, reaching the maximum effect in the range of 120–140 mM with EC50 of 107.9 mM ± 1.1 mM and 107.7 mM ± 2.1 mM, respectively (Figure 2d,e).

Therefore, the established model indicated that AM-induced swelling and lysis of RBCs dose-dependently correlate with [NH_4_^+^] in the buffer and might serve as markers of AM transport.

### 2.3. The eAE1 Exchanger and the RhAG Channel Are Functionally Connected

**Figure 3:**
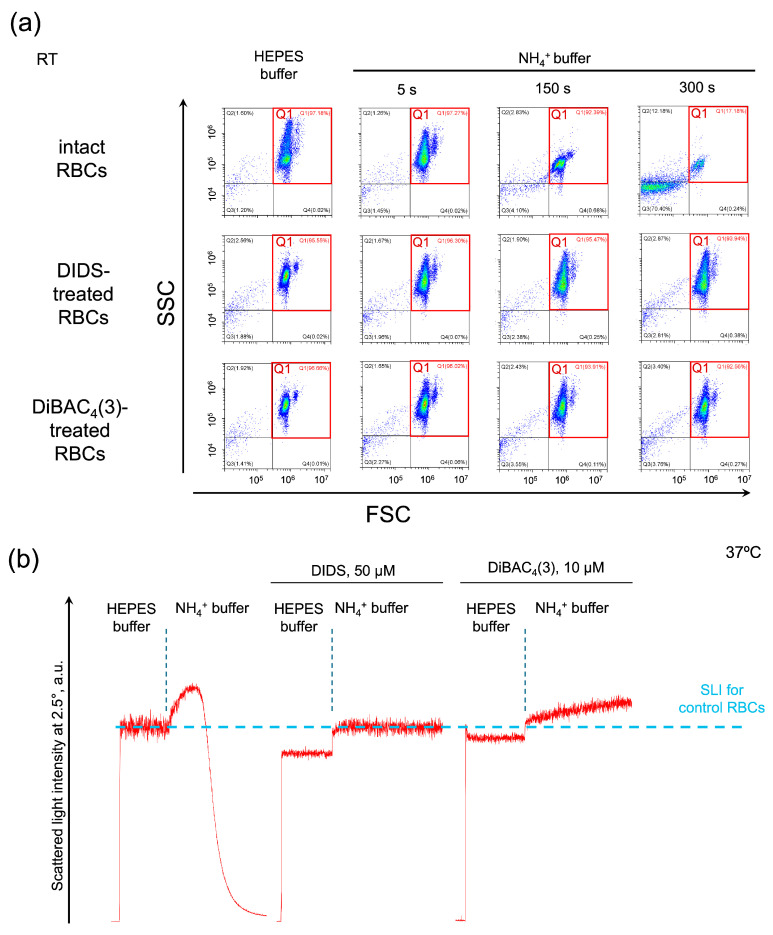
The human erythroid AE1 exchanger and RhAG channel are functionally connected. Representative AST for control cells and in conditions of eAE1 inhibition analyzed by flow cytometry ((**a**) one of 10) and laser diffraction ((**b**) one of 8). (**a**) Washed intact RBCs (10^6^ cells/mL), and DIDS or DiBAC_4_(3)-treated RBCs, suspended in HEPES buffer were analyzed by flow cytometry in forward scattering (FSC)/side scattering (SSC) coordinates for average size/granularity and hemolysis characterization. Next, RBCs were suspended in isotonic NH_4_^+^ buffer and immediately analyzed in the TIME mode for 5 additional min. FSC/SSC plots were then extracted at the indicated time points. FCS Express 7 Research was used for the visualization. The x–y scaling is preserved in all scatter-plots. (**b**) Washed intact RBCs (10^6^ cells/mL) or DIDS/DiBAC_4_(3)-treated RBCs were suspended in HEPES buffer and SLI was recorded for 2 min for control. Next, the RBCs were suspended in isotonic NH_4_^+^ buffer and registration continued for 2 min.

We hypothesized that the RhAG channel and eAE1 transporter are functionally coupled. If it is so, then inhibition of eAE1 will affect the AM-induced swelling and hemolysis. First, using flow cytometry, we analyzed the effects of eAE1 inhibition on AST. Washed RBCs were suspended in HEPES buffer for control, then in isotonic NH_4_^+^ buffer where almost complete hemolysis was observed within 3 min (Figure 3a, intact RBCs). The eAE1 inhibitors DIDS and DiBAC_4_(3) themselves led to a decrease in cell distribution width in the HEPES buffer, i.e., cells became more homogenous in size (Figure 3a, DIDS/DiBAC_4_(3)-treated cells suspended in HEPES buffer). In NH_4_^+^ buffer, cells were characterized by an increase in granularity width; however, either DIDS or DiBAC_4_(3), or both, completely prevented hemolysis (Figure 3a, DIDS/DiBAC_4_(3)-treated cells suspended in NH_4_^+^ buffer). 

Next, we examined the same reactions using laser diffraction. The control AST passed regularly with complete hemolysis within 2 min (Figure 3b, intact RBCs). eAE inhibition by DIDS triggered the decrease in LSI oscillations, indicating cell spherization (according to [51]) in HEPES buffer and a rapid and continuous increase in MCV up to 87 fL ± 4 fL upon suspending in NH_4_^+^ buffer (Figure 3b, DIDS); however, DIDS completely prevented hemolysis. eAE inhibition by DiBAC_4_(3) similarly led to cell spherization in HEPES buffer and to an increase in MCV up to 89 fL ± 3 fL upon suspending in NH_4_^+^ buffer (Figure 3b, DiBAC_4_(3)); however, DiBAC_4_(3) also completely prevented hemolysis. These data are in agreement with the flow cytometry data and elaborate on the absolute cell volume data in kinetics.

The presented data indicate that eAE1 inhibition prevents RBC swelling and lysis in the AST and suggested the involvement of eAE1 in AM transport.

### 2.4. Modulation of eAE1 Ligand Concentrations Significantly Affects Cell Swelling and Hemolysis in Isotonic AM Buffer

#### 2.4.1. Bicarbonate Restoration Dose-Dependently Increases the Rates of Cell Swelling and Hemolysis

As we have just shown, eAE1 and RhAG are likely to be functionally connected; therefore, the modulation of the concentration of eAE1 ligands, or their total absence, would affect AM-triggered cell swelling and hemolysis. In physiological conditions, eAE1 has two main high-affinity ligands, a bicarbonate ion, and a chloride ion, which are stated to be transported in an electrically silent way [6,19]. First, we analyzed the effects of changes in bicarbonate concentration on AM-induced cell swelling and hemolysis in isotonic NH_4_^+^ buffer. Thereto, we gradually restored HCO_3_^−^ concentration from 0 mM to physiological level of 25 mM in NH_4_^+^ buffer. 

**Figure 4:**
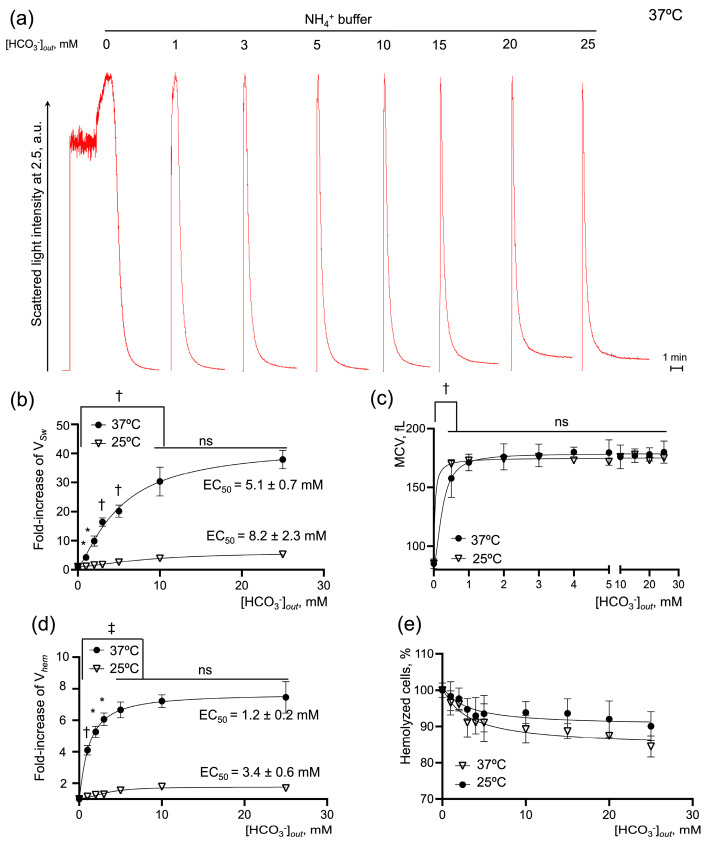
Restoration of [HCO_3_^−^] dose-dependently increases the rates of cell swelling and hemolysis in human RBCs. Washed RBCs (10^6^ cells/mL) were suspended in isotonic NH_4_^+^ buffer with HCO_3_^−^ in a range of 0 to 25 mM at 25 and 37 °C as indicated. The initial rate of cell swelling (V*_Sw_*) calculated from hemolysis curves increased dose-dependently with the addition of HCO_3_^−^. (**a**) Representative hemolysis curves from laser particle analyzer; (**b**,**c**) Quantification of EC_50_ for the cell-swelling rate and MCV max. (**d**,**e**) Quantification of EC_50_ for the maximal rate of hemolysis and % of hemolyzed cells. Data are presented as Mean ± SD, n = 10 for each temperature, one-way ANOVA, Leven’s test *p* > 0.05, Tukey HSD post hoc, *, *p* < 0.05, †, *p* < 0.01, ‡, *p* < 0.001, ns—not significant.

Bicarbonate potentiated the rates of cell swelling and hemolysis dose-dependently (Figure 4a). An increase in [HCO_3_^−^] to its physiological concentration of 25 mM enhanced the V*_Sw_* 49 ± 4-fold with EC_50_ of 5.1 ± 0.7 mM, reaching the plateau of the maximal rate at 10 mM at 37 °C (n = 10, *p* < 0.05). V*_hem_* increased 9.4-fold ± 0.5 with EC_50_ of 1.2 ± 0.2 mM and reached maximum at [HCO_3_^−^] 5 mM (n = 10, *p* < 0.05). At the same time, the increase in bicarbonate concentration did not have any significant effect on the MCV*_max_* or % of hemolyzed cells (Figure 4c,e). Additionally, to reconfirm that the process is predominantly governed by protein transport systems [52,53,54], we calculated the temperature coefficient Q_10_ for each response. If Q_10_ is around 1, then the reaction can be explained by the diffusion of ions, but if Q_10_ is greater than 2, the process is believed to involve large-scale protein conformational changes [53]. Therefore, we examined the effects of bicarbonate on AM-triggered cell swelling and hemolysis at a lower temperature of 25 °C. At 25 °C, [HCO_3_^−^] raised to 25 mM significantly potentiated V_Sw_ 6-fold ± 0.2 with EC_50_ of 8.2 ± 2.3 mM, V*_hem_* 0.6-fold ± 0.1 with EC_50_ of 3.4 ± 0.6 mM (compared to the appropriate controls, n = 10, *p* < 0.05) and had no significant effect on the MCV*_max_* or % of hemolyzed cells. Next, the Q_10_ was calculated at the saturation points for the rates of swelling and hemolysis that significantly differed at 25 °C and 37 °C. Both V*_Sw_* and V*_hem_* exhibited a high-temperature dependency, Q_10_ (V*_Sw_*) 4.53 ± 0.29 and Q_10_ (V*_hem_*) 2.99 ± 0.23. 

These data indicate that the rates of AM-triggered RBC swelling and hemolysis significantly depend on the bicarbonate concentration and suggest the importance of eAE1 in AM-triggered RBC swelling and hemolysis. Furthermore, we showed that the rates of AM-triggered RBC swelling and hemolysis under conditions of [HCO_3_^−^] modulation are highly sensitive to the temperature with Q_10_ above 2, which additionally confirmed that these reactions are mediated primarily by protein transporters involving changes in protein conformation and not facilitated or simple diffusion, as was often stated previously [31,55].

#### 2.4.2. Restoration of [Cl^−^] Dose-Dependently Potentiated AM-Induced Cell Swelling and Hemolysis

To establish whether the restoration of another major eAE1 substrate, chloride anion, concentration in Cl^−^-free buffers affected AM-induced cell swelling and hemolysis, we performed the AST in the modified isotonic NH_4_^+^ buffers containing [Cl^−^] in a range from 0 to 140 mM, in the presence or absence of bicarbonate in physiological concentration (25 mM). 

**Figure 5:**
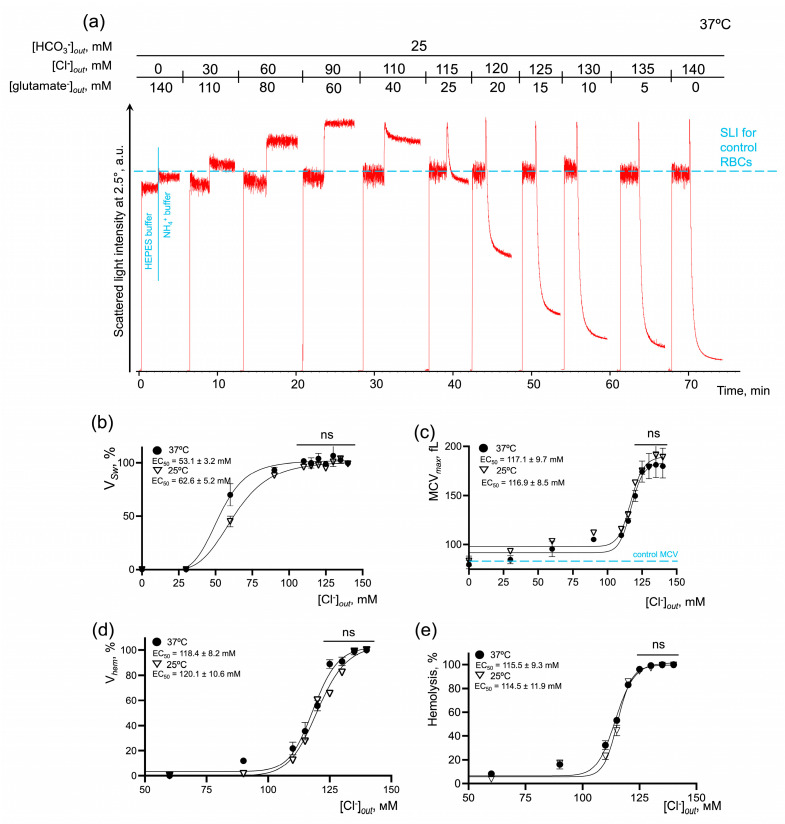
AM-induced RBC swelling and hemolysis are concentration-dependently potentiated by the [Cl^−^] restoration in constant bicarbonate concentration. (**a**) Representative experiment by laser diffraction, one of 10. Washed RBCs (10^6^ cells/mL) were suspended in HEPES buffer and SLI at a 2.5° angle was registered for 1 min for control. Then RBCs (10^6^ cells/mL) were suspended in isotonic NH_4_^+^ buffer and SLI registration continued until complete lysis occurred. Then RBCs (10^6^ cells/mL) were suspended in isotonic NH_4_^+^ buffers of the indicated constitution with gradual substitution of chloride anions for glutamate anions. The increase in SLI corresponded to cell swelling and the decrease in SLI corresponded either to cell spherization, or to hemolysis; (**b**) dose–response curve and EC_50_ quantification for V*_Sw_* at 25 and 37 °C; (**c**) dose–response curve and quantification of EC_50_ for MCV max at 25 and 37 °C; (**d**) dose–response curve and EC_50_ quantification for V*_hem_* at 25 and 37 °C; (**e**) dose–response curve and EC_50_ quantification for % of hemolyzed cells at 25 and 37 °C; Data are presented as Mean ± SD, n = 5 for each temperature, one-way ANOVA, Leven’s test *p* > 0.05, Tukey HSD post hoc, ns—not significant.

**Figure 6:**
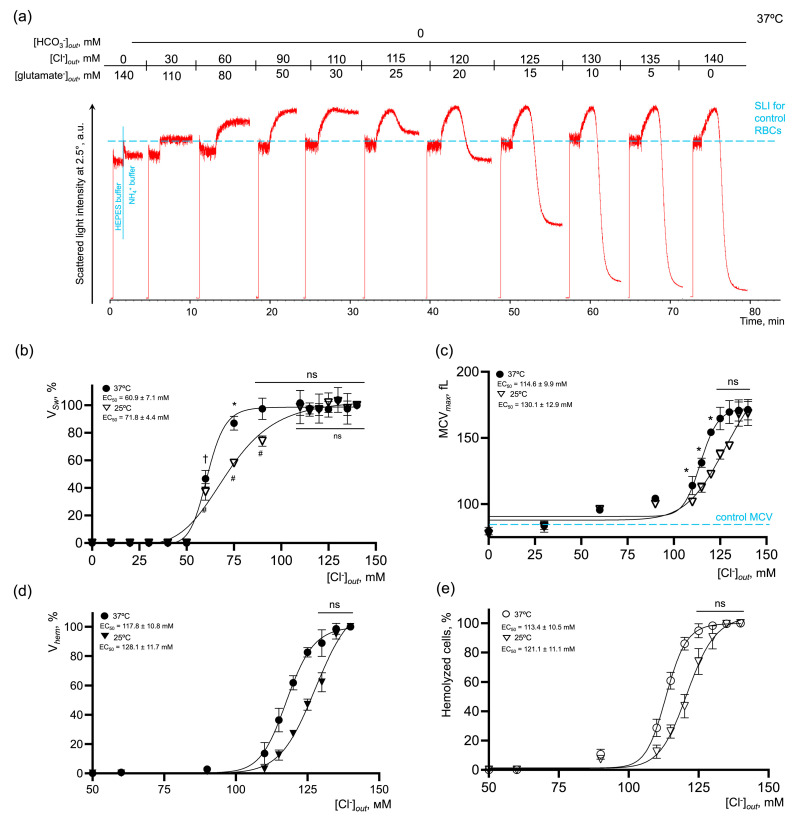
AM-induced RBC swelling and hemolysis are concentration-dependently potentiated by the [Cl^−^] restoration in the bicarbonate-free buffer. (**a**) Representative experiment by laser diffraction, one of 10. Washed RBCs (10^6^ cells/mL) were suspended in HEPES buffer and SLI at 2.5° was registered for 1 min for control. Then RBCs (10^6^ cells/mL) were suspended in isotonic NH_4_^+^ buffer and SLI registration continued until complete lysis occurred. Then RBCs (10^6^ cells/mL) were suspended in isotonic NH_4_^+^ buffers of the indicated constitution with gradual substitution of chloride anions for glutamate anions. The increase in SLI corresponded to cell swelling and the decrease in SLI corresponded either to cell spherization, or to hemolysis; (**b**) dose–response curve and EC_50_ quantification for V*_Sw_* at 25 and 37 °C; (**c**) dose–response curve and quantification of EC_50_ for MCV max at 25 and 37 °C; (**d**) dose–response curve and EC_50_ quantification for V*_hem_* at 25 and 37 °C; (**e**) dose–response curve and EC_50_ quantification for % of hemolyzed cells at 25 and 37 °C; data are presented as Mean ± SD, n = 5 for each temperature, one-way ANOVA, Leven’s test *p* > 0.05, Tukey HSD post hoc, *, *p* < 0.05, †, *p* < 0.01, #, *p* < 0.01, ns—not significant.

First, we showed that the substitution of chloride ions by glutamate had no negative effects on RBC viability or hemoglobin (Hb) (Figure S4). In NH_4_^+^ and HEPES buffers lacking chloride, the SLI oscillations were much lower than in the control cells (Figure 5a,c and Figure 6a,c), indicating that the cells most likely transformed from the discoid shape to a spherical shape. The increase in [Cl^−^] significantly and concentration-dependently amplified the rates of AM-induced cell swelling and hemolysis, and the % of hemolysis in the presence (Figure 5) or absence (Figure 6) of bicarbonate. The rates of cell swelling and hemolysis, as well as the dependence of the reaction on the temperature (Q_10_), were significantly lower in the bicarbonate-free buffers.

In bicarbonate-containing NH_4_^+^ buffer at 37 °C (Figure 5), the maximal effect on V*_Sw_* was reached at chloride concentrations in the range of 90–140 mM with EC_50_ 53.1 ± 3.2 mM. V*_hem_*, % hemolysis and MCV*_max_* reached the maximum values at [Cl^−^] over 125 mM with very close values of EC_50_ 118.4 ± 8.2 mM, 115.5 ± 9.3 mM, and 117.1 ± 9.7 mM, correspondingly. In bicarbonate-free NH_4_^+^ buffer at 37 °C (Figure 6), the kinetic parameters of NH_4_^+^-induced cell perturbations were very similar to those observed in bicarbonate buffers. Thus, the maximal effect on V*_Sw_* was reached at chloride concentrations in the range of 90–140 mM with EC_50_ 60.9 ± 7.1 mM. V*_hem_*, % hemolysis and MCV*_max_* reaching the maximum values at chloride concentrations over 125 mM also with close values of EC_50_ 117.8 ± 10.8 mM, 113.4 ± 10.5 mM, and 114.6 ± 9.9 mM, correspondingly. The absence of chloride anions in the buffer, i.e., inverted chloride gradient, in both bicarbonate and bicarbonate-free buffers, completely inhibited cell swelling and hemolysis even in 140 mM NH_4_^+^-containing buffer (Figure 5 and Figure 6). 

To analyze the temperature dependence of the reaction (Q_10_), the kinetic parameters of AM-triggered cell swelling and hemolysis were analyzed at 25 °C. In bicarbonate-containing NH_4_^+^ buffer, the maximal effect on V*_Sw_* at 25 °C was similarly reached at chloride concentrations in the range of 90–140 mM; however, to cause the half-maximal effect, more chloride (62.6 ± 5.2 mM) was required compared to at 37 °C. EC_50_ for V*_hem_*, % hemolysis, and MCV*_max_* at 25°C had almost similar values to those at 37 °C, i.e., there were no significant differences detected (Figure 5c–e). When the media lacked bicarbonate, the effects of temperature on the kinetics of AM-induced cell swelling and lysis were more pronounced compared to bicarbonate-containing NH_4_^+^ buffer. The maximal effect on V*_Sw_* was reached at chloride concentrations over 110 mM with EC*_50_* 71.8 ± 4.4 mM. V*_hem_*, % hemolysis and MCV*_max_*, similarly at 37 °C, reached saturation over 125 mM of chloride at 25 °C. At the same time, to cause the half-maximal effect, more ligand was required, EC_50_ 128.1 ± 11.7 mM, 121.1 ± 11.1 mM, and 130.1 ± 12.9 mM, correspondingly (Figure 6c–e). Next, we calculated the temperature coefficients Q_10_ for the rates of cell swelling and hemolysis at the saturation points. In the bicarbonate-containing buffer, the rates of cell swelling and hemolysis almost doubled from 25 °C to 37 °C with Q_10_ (V*_Sw_*) 2.53 ± 0.12 and Q_10_ (V*_hem_*) 1.94 ± 0.14, correspondingly (Figure 5b,d). In the bicarbonate-free buffer, the values for Q_10_ were as follows (V*_Sw_*) 2.75 ± 0.14 and V*_hem_* 2.05 ± 0.13 (Figure 6b,d).

These data showed that the rates of cell swelling and hemolysis and the percentage of hemolyzed cells are dose-dependently potentiated by the restoration of [Cl^−^]. The absence of chloride anions, i.e., an inverted chloride gradient, completely prevented cell swelling and hemolysis in NH_4_^+^ buffer at both 25 °C and 37 °C, indicating that extracellular chloride concentration or maintenance of chloride gradient are crucial. At the same time, we showed that the bicarbonate significantly amplified the rates of cell swelling and hemolysis in NH_4_^+^ buffers; however, its absence did not stop the reaction, which indicates that bicarbonate is probably a rate-limiting factor, whereas chloride concentration is most likely the driving force.

#### 2.4.3. Validation of the Laser Diffraction Data by Flow Cytometry

**Figure 7:**
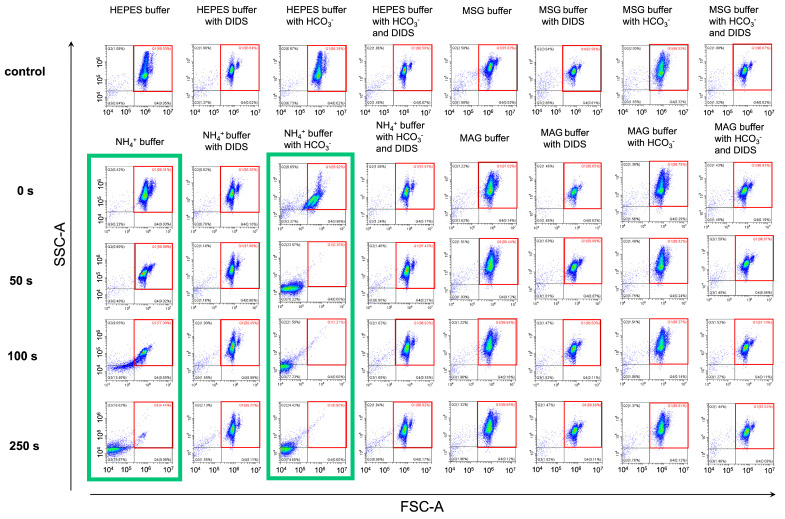
AM-triggered RBC swelling and hemolysis are inhibited under chloride-free conditions similar to the inhibition of eAE1. Washed RBCs (10^6^ cells/mL) were suspended in the indicated buffers and analyzed by flow cytometry in kinetics at RT. Q1—control gate, marked in the red square; Q2, Q3—lysis gates; Q4—cells with decreased granularity. The threshold was set to be minimal to correctly distinguish the events corresponding to lysis. Green boxes indicate the conditions favorable for hemolysis. A representative experiment of one donor RBC reaction to the AST under different conditions (one donor out of 10), the x–y scaling is preserved in all scatter-plots.

Next, to prove that our original method data correspond to the well-established flow cytometric approach, we performed the same experiments using flow cytometry at RT. RBCs were suspended in the following buffers: HEPES buffer (intact RBCs), NH_4_^+^ buffer (intact RBCs), NH_4_^+^ buffer (DIDS-treated RBCs), NH_4_^+^ buffer with HCO_3_^−^ (intact RBCs), NH_4_^+^ buffer with HCO_3_^−^ (DIDS-treated RBCs), MSG buffer (intact RBCs) and MAG buffer (intact RBCs) (Figure 7). In the kinetic studies involving the cell ensemble, we showed that NH_4_^+^-induced cell swelling and hemolysis are temperature dependent; therefore, here, in decreased temperature conditions (RT), each experimental period was extended to not less than 250 s.

In the NH_4_^+^ buffer (Figure 7, first green box), 77% of cells remained in the control gate Q1 (marked in red) at the 100 s point. At a point of 250 s, almost all cells moved from gate Q1 to gates Q2 and mostly Q3. In NH_4_^+^ buffer with HCO_3_^−^ (Figure 7, second green box), 15% of cells burst within the first seconds of the test, and full lysis was already achieved at the 50-s point. At the same time, more than 95% of cells remained in the control gate Q1 throughout the 250 s of the test in NH_4_^+^ buffer (DIDS-treated RBCs), NH_4_^+^ buffer with HCO_3_^−^ (DIDS-treated RBCs), MSG, and MAG buffers (with and without HCO_3_^−^). No hemolysis was observed in these conditions. These results were in good consistency with laser diffraction data.

Therefore, using both single-cell analysis and analysis of the cell ensemble, we obtained similar results in the AST model. Using both methods, we showed that eAE1 blocking as well as the lack of its primary substrate chloride inhibited AM-triggered swelling and lysis of RBCs. The advantages of flow cytometry are obvious, whereas the laser diffraction method enabled (i) a more precise and simple calculation of the cell volume changes and the rates of these changes, including characterization of hemolysis, and (ii) an exclusive constant temperature regulation.

### 2.5. Intracellular pH (pHi) in Inverted Chloride Gradient

eAE1 catalyzes the anion exchange between HCO_3_^−^ and Cl^−^ across the plasma membrane, thereby maintaining Cl^−^ concentration in a narrow range, facilitating the intracellular pH changes, and controlling cell morphology to an extent [4,6,56,57]. The inward gradient for Cl^−^ usually exceeds that for HCO_3_^−^, thus, this transporter catalyzes the net efflux of HCO_3_^−^, acting as a cell-acidifying mechanism or as an acid-loader [56,57]. To test how eAE1 function is affected in chloride-free experimental conditions during AST, we used pHi sensitive dye—BCECF-AM with the nigericin method [4]—for pHi calibration (see Section 4.7). 

**Figure 8:**
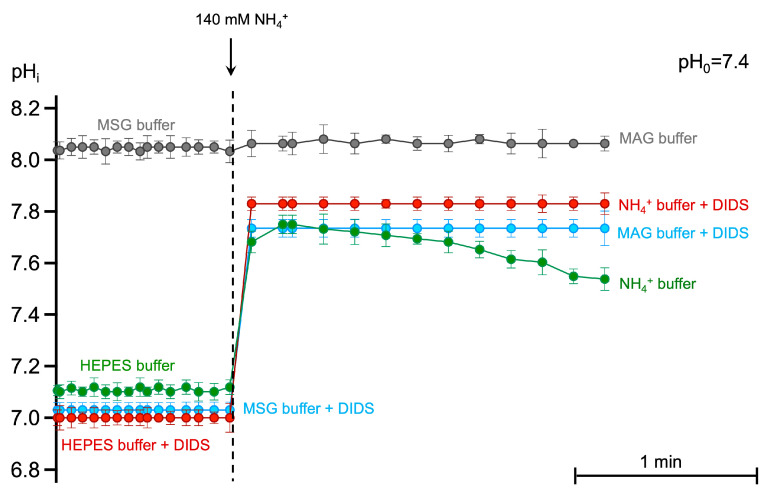
The lack of eAE1-mediated chloride gradient causes intracellular alkalization without compensatory secondary acidification. Washed RBCs (10^6^ cells/mL) stained with BCECF-AM (5 μM, 30 min, 37 °C) were suspended in the HEPES buffer (500 μL) of indicated constitution (300 mOsm kg H_2_O, pH_o_ 7.4) with immediate registration of BCECF fluorescence ratio at FITC-A/ECD-A in TIME mode over 60 s. Next, the stained cells were added to the 140 mM NH_4_^+^ containing buffers (300 mOsm kg H_2_O, pH_o_ 7.4), and registration of BCECF fluorescence was registered for additional 180 s. Where indicated, eAE1 was inhibited by the addition of DIDS (300 μM) in the corresponding buffer. The pHi calibration using the nigericin method was performed before each experiment. Each point on the graph is the Mean ± 95% CI of eight independent experiments. In each independent experiment, no fewer than 10 000 events were analyzed for BCECF fluorescence at each time point.

DIDS, as a strong acid with pKa of −3.21, lowered the pHi in both chloride-containing (Figure 8, red circles) and chloride-free (blue circles) conditions compared to control (green circles). In NH_4_^+^ buffer, pHi increased to 7.8 with the following compensatory eAE1-facilitated intracellular acidification (Figure 8, green circles after the dashed line). eAE1 inhibition by DIDS led to an increase in intracellular pH without compensatory acidification (Figure 8, red circles after the dashed vertical line). The same situation was registered for chloride-free MSG (Figure 8, grey circles before the dashed line) and MAG buffers (Figure 8, grey circles after the dashed line), where chloride gradient was lacking. However, the alkalinization degree in the case of eAE1 inhibition (NH_4_^+^ buffer + DIDS) and chloride gradient alteration (MSG and MAG buffers) significantly differed. No cell swelling was detected in MSG and MAG buffers, only cell spherization (Figure 5a and Figure 6a, Supplementary Figure S4). Considering the chemical properties of MSG and the buffering capacity of erythrocytes, we anticipated the intracellular pH to either remain stable at the control level or decrease slightly. However, unexpectedly, we observed significant intracellular alkalinization under these conditions (Figure 8, grey circles before the dashed line), MSG buffer). The alkalinization in MSG buffer was most likely triggered by the inversion of the band 3 function in chloride-free conditions: the lack of chloride anions in the media led to [Cl^−^]_in_ > [Cl^−^]_out;_ therefore, eAE1 was forced to act in the opposite direction as an acid-extruder. Moreover, low/lacking chloride solutions with an anion that is membrane impermeant also could reverse the hydrogen ion gradient [58], explaining the high pHi in MSG buffer. At the same time, the pronounced pHi increase in MAG buffer was most likely driven not only by the inverted eAE1 function (and hydrogen ion gradient) but also by the influx of NH_3_ (and not NH_4_^+^) solely (Figure 5a). which goes down the NH_3_ gradient and does not require band 3. In eAE1 inhibition, cell swelling was up to 20% higher compared to the appropriate control (Figure 3b, DIDS and DiBAC_4_(3)), which might be explained by the compensatory work of other transport proteins involved in volume and pHi equilibrium. 

These data indicate that chloride gradient is crucial for the influx of protonated form NH_4_^+^, whereas NH_3_ most likely enters the cell via simple diffusion causing the initial highest alkalinization.

### 2.6. eAE1 Activity Is Crucial for AM Import in Human RBCs

Previously, we showed that the AM-induced RBC swelling and hemolysis (which probably reflect AM transport) critically depended on eAE1 function. To prove that the analyzed kinetic parameters correlate with AM influx, we next established an indirect method of assessment of AM import according to the decrease in [NH_4_^+^] in the supernatant of RBCs incubated with NH_4_Cl in pathophysiological concentration. The AM concentration was determined using the enzymatic glutamate dehydrogenase spectroscopic method and the o-phthalaldehyde fluorometric approach for AM detection (see Section 4.8). RBCs were suspended in modified HEPES buffers with the addition of NH_4_Cl (800 µM). HEPES buffer with NH_4_Cl but without RBCs was used as a negative control for AM import.

**Figure 9:**
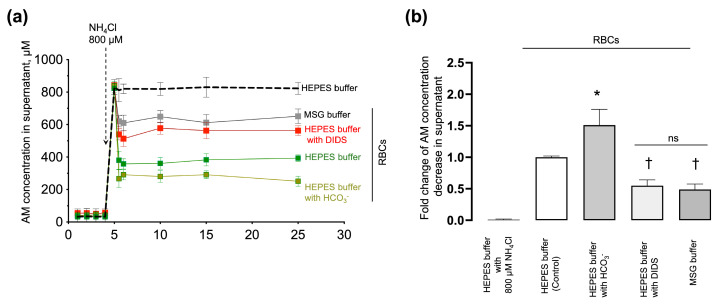
AM import correlated with the eAE1 function. (**a**) Washed RBCs (1.2 × 10^9^ cells/mL) were suspended in HEPES buffer (green squares), HEPES buffer with DIDS (red squares), HEPES buffer with HCO_3_^−^ (blue squares), and MSG buffer (grey squares) at 37 °C. Four aliquots of each probe were collected at 1, 2, 3, and 4 min time points, immediately centrifuged, and the supernatant was collected for further AM detection. Then NH_4_Cl (800 μM) was added to the samples, and aliquots were taken in (in min): 0.5, 1, 5, 10, 15, and 25 after the addition of AM, immediately centrifuged at 4 °C with a supernatant collection for a further AM concentration assay. A blank probe was run in HEPES buffer with 800 μM NH_4_Cl addition without RBCs for negative control (HEPES buffer, black dashed line). Data are presented as Mean ± SD, n = 6. One-way ANOVA for multiple comparisons, Leven’s test *p* < 0.05, Tamhane T2 post hoc, *, *p* < 0.05 compared to 800 μM NH_4_Cl addition; (**b**) quantification of the data from A. The decrease in AM concentration in HEPES buffer with RBCs (control) 30 min after AM addition was taken as 1. Data are presented as Mean ± SD, n = 6, one-way ANOVA, Leven’s test *p* < 0.05, Tamhane T2 post hoc, *, *p* < 0.05, †, *p* < 0.01 compared to AM decrease in the control, ns—not significant.

In the HEPES buffer without RBCs, [NH_4_^+^] remained constant (800 ± 17 μM) during the test period (Figure 9a, black dashed line). In a sample with intact RBCs (1.2 × 10^9^ cells/mL), [NH_4_^+^] decreased by 51% ± 9% (Figure 9a, green squares) in 1 min after NH_4_Cl addition. In a sample with DIDS-treated RBCs, [NH_4_^+^] in the supernatant decreased by 31% ± 5% (Figure 9a, red squares). In chloride-free buffer, [NH_4_^+^] decreased by 19% ± 2% (Figure 9a, MSG buffer, grey squares). Bicarbonate, in turn, potentiated AM import and [NH_4_^+^] in the supernatant decreased by 60% ± 5% (Figure 9a, khaki squares). 

Next, based on the experimental data on the decrease in AM in the supernatant after the addition of 800 µM NH_4_Cl (Figure 9b), we composed the balanced equation for the estimation of the theoretical intracellular [NH_4_^+^] concentration that corresponds to such AM decrease in the supernatant. According to the balanced equation (Supplementary Materials Section S3; Supplementary Figure S5), the estimated intracellular AM concentration relevant to such [NH_4_^+^] decrease in supernatant should be at least (in mM): 3.7 ± 0.4 (HEPES buffer, intact RBCs), 4.9 ± 0.6 (HEPES buffer with HCO_3_^−^, intact RBCs), 2.2 ± 0.2 (HEPES buffer, eAE1 inhibition), 1.4 ± 0.2 (MSG buffer, intact RBCs) (n = 5, *p* < 0.05). 

These data correlate with the kinetic studies using laser diffraction and flow cytometry and prove that cell swelling and hemolysis are triggered by AM influx.

## 3. Discussion

In humans, plasma AM level is maintained in the narrow range from 12 to 60 μM and an increase may lead to serious health issues, mostly neuropsychiatric and neurological, such as convulsions, seizures, brain edema and damage, and even coma and death [20,33]. The intracellular AM concentration in RBCs exceeds the plasma concentration by three [32] to seven times [38]. In AM detection kits, it is strictly prohibited to use samples with even a sign of hemolysis because AM concentration increases markedly in the presence of ruptured cells. Indeed, it has been reported that upon storage, in either human [34,59,60], feline [61,62], or canine [63] blood units, AM concentration increased up to 6-fold or up to 450 µM in the preservative solution; therefore, the transfusion of unwashed blood units may even be harmful to those patients with hyperammonemia symptoms [59]. Taken together, all these data contribute to the assumption of the RBC capacity of AM accumulation. At the same time, our data suggest that RBCs trap the excessive NH_4_^+^ from the media indicating that NH_4_^+^ is imported into human RBCs against its gradient (Figure 9 and Supplemental Figure S5). The possibility of such transport by RhAG even by simple diffusion of NH_3_ was previously doubted precisely because of the unfavorable AM gradient [64]. Thus, there should be some auxiliary force (coupled transport, electrochemical gradient, etc.,) that oversees the NH_4_^+^ influx. 

AM transport cannot be registered directly; therefore, the most used method for AM flux analysis is microelectrode detection of the AM-induced pH_i_ changes [39] or AM-induced changes in pH_i_ analyzed with pH-sensitive fluorophores. However, such an approach is highly relative as the maintenance of pHi is a very fast and complex process involving different proteins. Studies of the role of RhAG were conducted mainly in heterologous models transfected with RhAG, such as *Xenopus* oocytes [42,65] or HeLa cells [66], and NH_3_ influx was detected as the slope of the initial alkalinization, and NH_4_^+^ influx as the slope of secondary acidification. However, in the mentioned studies, the initial ratio of AM_intracellular_/AM_plasma_ which is at least 3/1 was not considered. Therefore, the described simple diffusion of NH_3_ which is stated to be the first step to equilibrium would barely occur at physiological conditions due to the AM gradient [64]. Several studies were conducted on Rh_null_ RBC ghosts which exhibited a slower NH_3_ influx [39,40]; however, the ghosts are already ruptured cells and would not react to different conditions in the same way as intact RBCs. pH_i_ data were quite controversial, depending on the RhAG expression model and on the presented base and acid extruders. Previously, we [29] and others [4] showed that the relationship between pH_i_ and cell volume changes is much more complicated than it seems at first sight. Accordingly, we showed that in the NH_4_^+^ buffer, the maximum cell volume is reached even after secondary intracellular acidification is initiated [29]. Our new data on eAE1 presented in this paper also showed that, to correctly assess Rh glycoprotein-mediated AM transport, it is very important to consider eAE1 expression and its proper function in the model. Thus, to date, the nature of the transporting substrate remained questionable [22,40], and the driving force of AM influx has never been discussed. Consequently, to confirm the physiological relevance of AM transport by RBCs, these data were not enough.

As pH_i_ data may be controversial and there is no direct method for AM flux registration, in this study, we established the original method for the analysis of AM transport according to AM-triggered cell swelling and hemolysis. To characterize the kinetics of AM-containing buffer effects on human RBCs, we used two approaches based on light scattering including single-cell analysis by flow cytometry and laser diffraction analysis of cell ensemble. Using the advantages of these two approaches, we showed that RBCs transport AM only in conditions favorable for eAE1 activity as an acid-loader. eAE1 inhibition either by DIDS (the well-established eAE1 inhibitor), or DiBAC_4_(3) (one of the most selective eAE1 inhibitors reported to date [67,68]) completely prevented hemolysis in NH_4_^+^ buffer (Figure 3). Previously, the possibility that RhAG might be a CO_2_ channel and that DIDS is a selective inhibitor for RhAG was reported [67,68,69]. Our new results indicate that AM-induced RBC swelling and lysis and the corresponding AM transport are modulated by eAE1 main ligand concentration changes in a dose-dependent manner (Figure 4, Figure 5 and Figure 6). Adjusting [HCO_3_^−^] to the physiological values enhanced the rates of cell swelling almost 50-fold and hemolysis almost 9-fold at 37 °C (Figure 4). Such potentiation of the effect agreed with the data on eAE1 affinity to HCO_3_^−^ which was shown to be four times higher than that of chloride [7]. The restoration of chloride concentration to the physiological values also potentiated cell swelling and hemolysis. RBC shrinkage in conditions of complete replacement of extracellular Cl^−^ in 140 mM NH_4_^+^-containing buffer, i.e., in the absence of chloride gradient (Figure 5, Figure 6 and Figure 7, Supplementary Figure S4) were observed in both MSG and MAG buffers. Stilbenes as DIDS were shown to occupy the external binding site of eAE1, inducing a conformational change that locks the protein into an outward-facing state [6,70], thus preventing almost all Cl^−^ binding. Therefore, these results are in accordance with the already well-established mechanism of the eAE1 function. Thus, our data indicate that bicarbonate most likely serves as the limiting factor, regulating the rate of the reaction. Chloride, in turn, is vital for RBC swelling and hemolysis in NH_4_^+^ buffer, and is the switcher, or driving force, of the reaction, as in its absence no swelling or lysis occurs (Figure 5, Figure 6 and Figure 7). 

The temperature dependencies of the rates of AM-triggered cell swelling and hemolysis indicate that these responses are highly sensitive to the temperature (Q_10_ between 2 and 3), additionally confirming that AM-induced swelling and lysis of RBCs are mediated primarily by protein transporters and not mediated by facilitated or simple diffusion [52]. In chloride restoration, the effects of a temperature increase from 25 °C to 37 °C on the rates of cell swelling and hemolysis were much less pronounced (Figure 1 and Figure 2b,d) than in bicarbonate restoration (Figure 5b,d) which might be explained by the difference in these ligands specificity to eAE1 [7].

It should also be noted that in the range of AM concentrations up to 80 mM, the cells swelled from 85 fL to 95 ± 5 fL (Figure 5c), indicating that the capacity of RBCs to transport AM without altering the RBC integrity is very high. According to the conducted kinetic studies and examination of MCV*_max_* changes, it is most likely that RBC swelling triggered by AM is characterized by two phases (Figure 2c, Figure 5c and Figure 6c): initial phase of slight cell swelling to 95 ± 5 fL (0–80 mM) and second phase associated with fatal cell swelling and rupture (90–140 mM); however, the elucidation of the exact mechanisms merits future examination. 

We also showed that RBCs can trap and accumulate AM (Figure 9, Supplementary Figure S5) in conditions of excessive extracellular AM concentration. Importantly, consistent with flow cytometry and laser diffraction data, the AM import was highly diminished when eAE1 was inhibited, or with a lack of chloride anions in the extracellular media. 

Thus, our data on the kinetics of AM-induced cell swelling and hemolysis and AM import by RBCs in hyperammonemic media independently indicated that RBC swelling and hemolysis in AM-containing media occur due to the coupled function of the ammonium channel RhAG and erythroid AE1 which accounts for the maintenance of the chloride gradient.

**Figure 10:**
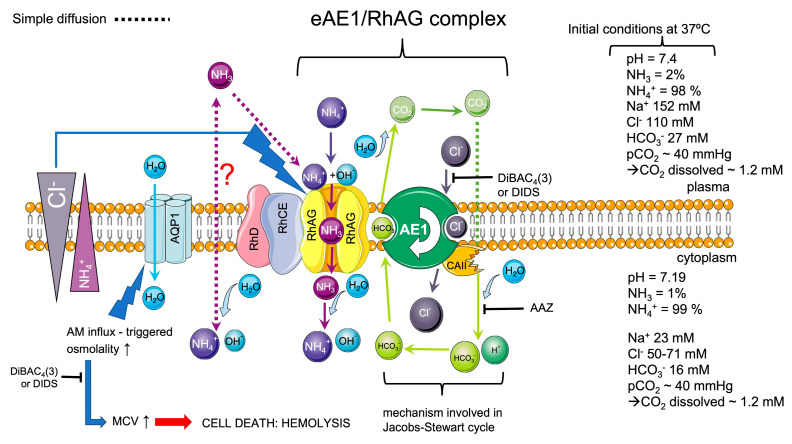
The scheme of the proposed mechanism of the teamwork of eAE1 exchanger and RhAG ammonium channel. The initial conditions (on the right) are marked according to [7,47,77,78,79]. Assuming these conditions and considering the intracellular/extracellular AM gradient [38,59], basically, the charged ammonium has no way inside the cell unless the transport is active. The influx of uncharged ammonia is highly dubious and would theoretically occur only if the NH_3_ gradient is favorable, which depends on pH, pKa, and consequently on plasma NH_3_ concentration. As we showed (Figure 10), RBCs can trap AM (either protonated or neutral) against the gradient, which in turn triggers the cascade of events, including, in order of appearance, (a) pHi increase [4,39], (b) osmolality increase caused by the coupling chloride influx to compensate the increased pHi (net NH_4_Cl influx), (c) corresponding AQP1-mediated water influx [42,43], and (d) volume increase. According to our experiments, the coupling force which affords AM transport against the gradient is the electrochemical chloride gradient mediated by eAE1.

According to the 3D modeling and crystallography of the Amt-transporter, which is homologous to RhAG, it was proposed earlier [71] that NH_4_^+^ loses the hydration shell and deprotonates in the perimembranous region of the RhAG transporter; then in the uncharged form of NH_3,_ it passes the hydrophobic pore into the cell, and on the inner side of the channel, NH_3_ is reprotonated to the hydrated membrane-impermeable NH_4_^+^ form. The AM influx triggers a pHi increase, which should be compensated for by the acid-loaders as eAE1 by intracellular HCO_3_^−^ extruding for extracellular Cl^−^. These processes are characterized by the net influx of NH_4_Cl, which triggers an increase in osmolality and the corresponding influx of H_2_O through the lipid bilayer, or/and mediated by AQP1 [43], which leads to cell swelling until a new osmotic equilibrium is reached or until the cell undergoes lysis, as in our model reaction (Figure 1). However, if one of the ions involved in the exchange is H^+^ or OH^−^, then the symport of the transported substrate with H^+^ can hardly be distinguished from the antiport with OH^−^. We previously suggested that such AM trapping and accumulation against the gradient could be driven by the maintenance of an alkali pH in the perimembranous region of the RhAG channel, or by the maintenance of eAE1-facilitated pH gradient across the membrane at both sides of the channel, or even both [29,72]. However, our new data (Figure 7, Figure 8 and Figure 9) suggest that eAE1 plays the role of the coupling force via the maintenance of the chloride gradient and this energy is used to pass AM into the cell against the gradient (Figure 10). These results are consistent with the proposition that Cl^−^ probably plays a more important role in physiology than is generally appreciated [73,74,75]. The chloride/bicarbonate exchange mediated by eAE1 is one of the main processes which are in charge of carbon dioxide transport through the Jacobs–Stewart cycle [18]; therefore, its involvement in AM transport indicates that AM transport may be another fundamental function for RBCs. Here, we proposed the mechanism (Figure 10) according to which it is likely that eAE1 interacts with RhAG as a transporter would interact with an ion channel, forming a so-called ion channel-transporter (chansporter) complex [76]. However, these data merit future examination.

## 4. Materials and Methods

### 4.1. Reagents and Working Buffers

To analyze the AM-triggered RBC swelling and lysis (AM stress test, AST), we used a modified NH_4_^+^ medium in which chloride ions were substituted with glutamate ions. The buffers were isotonic (300mOsm kg H_2_O), had pH of 7.4, and had constant (in mM): EGTA, 2, Glucose, 5, and HEPES, 10. Other constituents varied as indicated (in mM): HEPES buffer (NaCl, 140; KCl, 2, MgCl_2_, 2); NH_4_^+^ buffer (NH_4_Cl, 140, KCl, 2, MgCl_2_, 2); HEPES buffer with HCO_3_^−^ (NaCl, 120; NaHCO_3_, 25; KCl, 2, MgCl_2_, 2); for NH_4_^+^ buffer with HCO_3_^−^ (NH_4_Cl, 120; NaHCO_3_, 25; KCl, 2, MgCl_2_, 2); for monosodium glutamate (MSG) buffer (MSG, 140); for MSG buffer with HCO_3_^−^ (MSG, 115; NaHCO_3_, 25); for monoammonium glutamate (MAG) buffer (MAG, 140); for MAG buffer with HCO_3_^−^ (MAG, 115; NaHCO_3_, 25). Where indicated, NH_4_^+^ buffer and NH_4_^+^ buffer with HCO_3_^−^ were equimolarly matched to achieve the desired bicarbonate concentration; NH_4_^+^ buffer (NH_4_^+^ buffer with HCO_3_^−^) and MAG buffer (MAG buffer with HCO_3_^−^) were equimolarly matched to adjust to the desired chloride concentration in the absence or presence of bicarbonate. All buffer components, Calcein-AM (C-AM, cat. 56496), *tert*-Butyl hydroperoxide (*t*-BOOH, cat. 8.14006), and 4,4′-diisothiocyano-2,2′-stilbene disulfonate (DIDS, cat. D3514) were purchased from Sigma Aldrich (cat. D3514, Munich, Germany). BCECF-AM (cat. B1170) and bis(1,3-dibutylbarbituric acid) trimethine oxonol (DiBAC_4_(3), cat. B438), we obtained from Invitrogen (Carlsbad, CA, USA). The osmolality of the buffers was monitored by Osmomat 030 (Gonotec GmbH, Berlin, Germany).

### 4.2. RBC Preparation

Human blood was obtained from healthy volunteers, who did not take any medication at least 10 days prior to the experiments, after giving informed consent. The blood draw was performed according to the Sechenov Institute of Evolutionary Physiology and Biochemistry of the Russian Academy of Sciences guidelines and the Declaration of Helsinki. Studies using human RBCs were approved by the Ethics Committee of IEPhB RAS (protocols no. 3–03 from 2 March 2021, and no. 1–04 from 7 April 2022). RBCs were obtained by centrifugation of whole blood at 350× *g* (centrifuge CM-6M, ELMI Ltd., Riga, Latvia) for 7 min at room temperature (RT). Washed RBCs were prepared by two-fold centrifugation of the pelleted RBCs at 400× *g* (centrifuge 5810R, Eppendorf, Hamburg, Germany) in HEPES buffer for 3 min, RT. Washed RBCs were resuspended in HEPES buffer and adjusted to 0.5 × 10^9^ cells/mL (corresponding to hematocrit, HCT, 4–5%) or 1.2 × 10^9^ cells/mL (corresponding to HCT 10%) as indicated. 

### 4.3. Washed Platelet Preparation

Human platelets were prepared as previously described [80,81]. Briefly, to generate platelet-rich plasma (PRP), citrated whole blood with the preliminary addition of EGTA (2 mM) was prepared as described above. PRP was collected in an ACD solution (in mM: citric acid, 12; sodium citrate, 15; D-glucose, 25) and centrifuged (200× *g*, 10 min; RT). To reduce leukocyte contamination, PRP was resuspended in a 1:1 ratio in CGS buffer (in mM: NaCl, 120; trisodium citrate, 12.9; D-glucose, 10; pH 6.5) and centrifuged (240× *g*, 10 min). After that, the supernatant was centrifuged (10 min, 430× *g*), pelleted platelets were washed in CGS buffer and resuspended in HEPES buffer with 1 mM CaCl_2_ and adjusted to the final concentration of 1 × 10^8^ platelets/mL for flow cytometry. The washed platelets then rested in the water bath (15 min, 37 °C) until the experiments.

### 4.4. Inhibition of eAE1

To inhibit eAE1, RBCs (0.1 × 10^9^ cells/mL) were incubated with specific inhibitor DIDS (for preincubation 50 μM, 5 min, RT; or for immediate inhibition 100 μM, 1 min, 37 °C) [4], or oxonol derivative DiBAC_4_(3) (5 μM, 5 min, 37 °C) [68,69,82,83].

### 4.5. Analysis of Intracellular Esterase Activity

Calcein-AM was used for the evaluation of cell esterase activity and corresponding cell vitality. The RBCs (0.5 × 10^9^ cells/mL) were incubated with calcein-AM (5 μM, 30 min, 37 °C) with the following calcein fluorescence registration either by the CytoFLEX flow cytometer (BeckmanCoulter, Brea, CA, USA) at the fluorescence light sensor 1 (FL1) or by the CLARIOstar Plus microplate reader (BMG LABTECH GmbH, Ortenberg, Germany) with excitation at 488 ± 14 nm and emission registration at 530 ± 30 nm. 

### 4.6. Spectral Analysis of Hemoglobin (Hb) Species

Absorption spectra of Hb species obtained from intact cells were registered by spectrophotometer SPECS SSP-715-M (Spectroscopic systems, Ltd., Moscow, Russia) and microplate reader CLARIOstar Plus in the wavelength range of 400–700 nm with a step size of 1 nm at 37 °C. Washed RBCs (10^6^ cells/mL) were incubated in the indicated buffers at the indicated temperatures and then the absorption spectra of the whole cells were scanned every 0/1/3/5/15 min. For negative control, we used RBCs under strong oxidative stress induced by tert-Butyl hydroperoxide (t-BOOH, 1 mM, 1 h). To analyze the free Hb spectra, RBCs were hypotonically lysed in MilliQ dH_2_O (50 μL RBC 0.5 × 10^9^ cells/mL in 2000 mL MilliQ dH_2_O) or NH_4_^+^ buffer (50 μL RBC 0.5 × 10^9^ cells/mL in 2000 mL NH_4_^+^ buffer), as indicated.

### 4.7. Intracellular pH (pH_i_) Analysis

Washed RBCs were prepared as mentioned above and pre-incubated with pH-sensitive dye BCECF-AM (5 µM, 30 min, 37 °C). Stained cells were incubated in different media as indicated. Intracellular pH changes were monitored continuously for 1.5–2 min. The intensity of BCECF fluorescence was measured by CytoFLEX flow cytometer (Beckman Coulter, Brea, CA, USA), via the ratiometric approach, according to the manufacturer’s protocol. The fluorescence of BCECF was excited at 488 nm, emission was registered at 530/40 nm (FL1 or FITC-A) and 613/20 nm (FL3 or ECD-A), and the ratio of emissions registered in channel FL1 to emissions registered in channel FL3 (RATIO FL1/FL3) was used for characterization of pHi changes. For calibration, the standard nigericin technique was used [4].

### 4.8. Characterization of AM Transport by Laser Diffraction

To monitor AM-triggered changes in RBC volume (MCV), the rate of initial cell swelling (V*_Sw_*, 10 s after the AST start), the maximal hemolysis rate (V*_hem_*), and the percent of hemolyzed cells (% hemolysis) in the AST model, laser diffraction method (laser microparticle analyzer LaSca-TM, BioMedSystems Ltd., Saint Petersburg, Russia), adapted for cell physiology, was used, according to [29,51,84,85,86,87]. The intensity of scattered light (scattered light intensity, SLI) for the control cells suspended in the HEPES buffer and for the cells in the AST model suspended in the NH_4_^+^ buffer was continuously detected by forward scattering in the range of 1 to 12° angles. According to the laser diffraction principles, cell swelling is characterized by an increase in SLI at 1–6° angles or a signal decrease in the range of 6–12° angles, hemolysis is characterized by a decrease in SLI throughout the detectors from 1 to 12° [88]. To assess EC_50_ for kinetic parameters against changes in AM, bicarbonate, or chloride concentrations in the AST model, we equimolarly substituted basic NH_4_^+^ buffer constituents by adjusting to the desired concentrations of ions. To control the accuracy of MCV calculation by the original software LaSca v.1498 of the LaSca-TM laser particle analyzer, MCV data from the Medonic-M20 hematological counter (Boule Medical A.B., Stockholm, Sweden) were used.

### 4.9. Evaluation of AM Import by Spectro- and Fluorometry

The import of AM under different conditions (eAE1 inhibition, chloride, and hydrocarbonate substitution) was analyzed according to the residual AM concentration in the supernatant after RBC incubation with 800 μM NH_4_Cl. RBCs (1.2 × 10^9^ cells/mL) were suspended in 2 mL of the indicated buffer at 37 °C. Then 4 aliquots (controls 1–4) of 100 µL were taken out of the sample, centrifuged and the supernatant was taken for further assessment of AM concentration. NH_4_Cl (800 µM) was then added to the sample and 100 µL aliquots were taken 30 s, 1 min, 5 min, 10 min, 15 min, and 25 min after AM addition. Each aliquot was immediately centrifuged, and the supernatant was separated for further assessment of the AM concentration. To assess AM concentration, we used spectrophotometric (Ammonia Assay Kit AAK0100, Sigma Aldrich, Germany) and fluorometric (Ammonia Assay Kit MAK310, SigmaAldrich, Germany) approaches according to the manufacturer recommendations with small modifications. Supernatants were analyzed using microplate reader CLARIOstar Plus (BMG LABTECH GmbH, Ortenberg, Germany). For the calibration curve, NH_4_Cl and (NH_4_)_2_SO_4_ standards were used.

### 4.10. Data Analysis

Laser diffraction data were analyzed using the original software of the LaSca-TM laser particle analyzer—LaSca v.1498 (BioMedSystems, Ltd., Saint Petersburg, Russia). Flow cytometry data were analyzed using CytExpert 2.4 for CytoFLEX cytometer (BeckmanCoulter, Brea, CA, USA) and by FCS Express Flow 7 (De Novo Software, Pasadena, CA, USA). Smart Medical Art presets (Les Laboratoires Servier, Suresnes Cedex, France) were used for RhAG/eAE1 chansporter complex visualization. The differences between the groups were analyzed by GraphPad Prism v.9 (GraphPad Software, San Diego, CA, USA). Data are presented as Means ± SD. The normal distribution was tested with the Kolmogorov–Smirnov normality test. For multiple comparisons, one-way ANOVA followed by Tamhane T2 (if Leven’s test *p* < 0.05), or Tukey HSD post-hoc (if Leven’s test *p* > 0.05) was used. For all tests, values of *p* < 0.05 were considered statistically significant.

## 5. Conclusions

The data reported here support the hypothesis that RBCs are able to trap AM [34,38,59] and suggest the important role of RBCs as a non-hepatic mechanism for controlling the levels of such nitrogen-related metabolites as ammonia/ammonium. Perhaps the interactions between eAE1 and other transport proteins or enzymes such as CAII are much more complicated and physiologically important than initially anticipated.

Excessive AM concentration, which is usually detected in patients with liver and kidney failure [89], diabetes mellitus [38], or in those who are under strenuous exercise [33,90], might also lead to oxidative stress and/or be solely a cause of the intravascular hemolysis or increased RBC microparticle shedding [91,92], thus contributing to the progression of comorbid factors such as anemia. Moreover, such complications as brain edema directly depend on the AM concentration in blood and its fluxes to the brain where elevated AM concentrations could alter the essential neuronal glutamate–glutamine cycle [93]. Therefore, a study of AM transport mechanisms becomes especially important in the hyperammonemia high-risk groups, as an understanding of AM transport regulation and mechanisms holds the potential to develop mechanistic and targeted treatments for hyperammonemia. Thus, the ability of RBCs to import and trap ammonium/ammonia is already used in erythrocyte-bioreactors that are artificially loaded with enzymes for increased utilization of excessive plasma ammonium [94,95]. Moreover, drugs which might alter the mechanism of AM trapping by RBCs should be administrated carefully so as not to disturb the narrow AM homeostasis. Thus, as the chloride gradient is vital for AM transport, chloride transport inhibitors such as tamoxifen, NPPB (5-nitro-2-(3-phenylpropylamino)-benzoic acid), or niflumic acid should be administrated with caution [96,97]. 

According to the proposed model, except for the general players, RhAG and eAE1, carbonic anhydrase II (CAII), the activity of which was shown to be tightly connected to the eAE1 function [7,73,98,99], might also be considered as a target, as there are several reports on CAII inhibitor (acetazolamide, topiramate, etc.,)-associated hyperammonemia [100,101,102,103] and reduced function [104]. Indeed, our preliminary data on CAII inhibition in the AST model showed that the rates of cell swelling and hemolysis were significantly reduced in RBCs treated with acetazolamide (AAZ, Supplementary Figure S6). However, these assumptions need further investigation. 

In conclusion, in this study, we developed an original method for the indirect assessment of AM transport in RBCs according to AM-triggered cell swelling and hemolysis. The inhibition of eAE1 and modulation of eAE1-ligand concentration affected AM transport and the corresponding import, suggesting that eAE1 is involved in the process. Our data indicate that the eAE1-mediated chloride gradient is required for AM transport against the AM concentration gradient. Taken together, our data reveal the new player in AM transport in RBCs.

## Data Availability

The data underlying this article will be shared after reasonable request is made to the corresponding author.

## Supplementary Materials

The following supporting information can be downloaded at: https://www.mdpi.com/article/10.3390/ijms25137390/s1. References [105,106,107,108,109,110,111,112,113,114] are cited in Supplementary Materials.
