# Supplementary material for: Chloride Gradient Is Involved in Ammonium Influx in Human Erythrocytes
Source: Int J Mol Sci. 2024 Jul 5;25(13):7390. doi: 10.3390/ijms25137390 (PMC11242273; doi:10.3390/ijms25137390)
Supplement: Supplementary file 1 [file ijms-25-07390-s001.zip › ijms-3003399-supplementary.pdf]

SUPPORTING MATERIAL

***1 Characterization of absolute cell volume changes by laser diffraction*.**

1.1 Flow cytometry for RBC volume sensing in dynamics

Determination of the absolute cell volume and cell volume changes is a challenging task [1, 2]. Flow cytometry can be applied as a tool, and volume can be detected according to forward scattering (FSC), or side scattering (SSC) and autofluorescence, and even violet SSC (VSSC), however only relatively [3]. Flow cytometry basically uses single cell analysis with data averaging within the collection angles of light scattering, which may vary significantly between the cytometers, e.g., from 1 to 19° in Navios; 1 to 100° in CytoFLEX; below 104° in FACSCalibur, FACSCanto, FACSVerse, Gallios [4, 5]. However, in conditions of kinetic determination, such averaging can cause inaccurate results or misinterpretation. The nonspherical shape of RBCs, according to the attitude position in the flow, will cause the light to be scattered differently at narrow and wide detectors thus, the initial cell volume increase might be incorrectly detected by flow cytometry as volume decrease [3, 6].

To check whether the flow cytometric method (CytoFLEX, BeckmanCoulter, USA) is appropriate for accurate kinetic registration of volume changes induced by osmotic gradients (osmotic stress, ammonium stress test), we used a basic osmotic fragility test in the range of osmolalities from 300 to 92 mOsm/ kg H_2O. Generally, RBCs under hypoosmotic stress swell due to the H_2O influx related to the osmotic gradient, and if osmolality is less than 150 mOsm/ kg H_2O – burst or undergo lysis [6, 7].

CytoFLEX has a high sensitivity for fluorophores, however, FSC on this cytometer is not classical small or low-angle light scattering, but a comparative signal analysis – axial light loss detection[5]. In our experiments at both FSC and SSC, CytoFLEX detected the well-established [3, 6, 8] volume increase at 200, 150, and 133 mOsm/kg H_2O as volume decrease (Supplement Figure S1B), even though the remaining events count was correct. Next, we examined whether using another light scattering-based device, the laser particle analyzer LaSca, which examines the ensemble of cells separately at different scattering angles[9], is appropriate for our model. Laser diffraction detected the rise in volume correctly (Supplement Figure S1D), and the light scattering intensity significantly differed at narrow (Supplement Figure S1C, 1° and 2.5°) and wide (Supplement Figure S1C, 12°) scattering angles. To overcome this issue, next, we developed an original method for scattering intensity data conversion into cell sizes (see section *1.2)*.

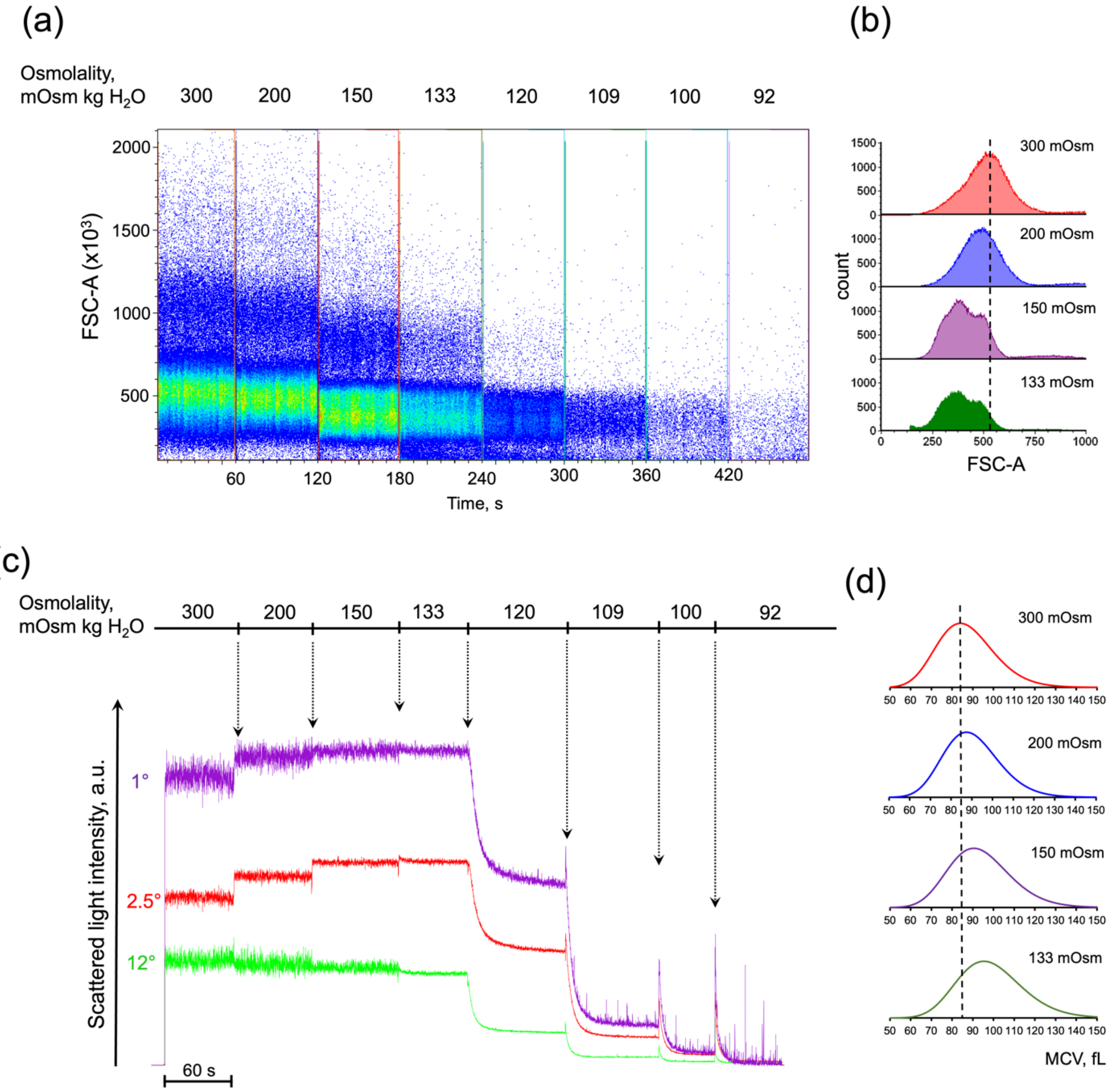

Supplementary Figure S1. Limitations of flow cytometry method for cell volume estimation in kinetics. (a) - To assess the flow cytometer sensitivity in dynamic RBC volume changes estimation, we performed a classical osmotic fragility test in the range of osmolalities from 300 to 92 mOsm/ kg H_2O. The representative pseudo color plot of FSC-A in linear coordinates against time, one of 3 experiments. Washed RBCs (10^6 cells/mL) were suspended in HEPES buffer (1 mL, pH 7.4, RT, 300 mOsm/kg H_2O), and the sample was analyzed for 60 s. Then, RBCs were suspended in HEPES buffer with decreased osmolalities as indicated, incubated for 1 min, vortexed, and FSC was registered for 60 s; **(b)** – The gates for each osmolality were analyzed using FCS Express 7 and the corresponding FSC distributions were depicted; (**c)** – To assess the laser particle analyzer LaSca sensitivity for osmolyte-induced volume changes we performed the classical OFT according to [10]. Washed RBCs (10^6 cells/mL) were suspended in HEPES buffer (1 mL, pH 7.4, 37°C, 300 mOsm/kg H_2O), and the corresponding to control scattered light intensity (SLI) was registered. Next, the distilled H_2O (in uL: 500, 500, 250, 250, 250, 250) was added to the test tube with the corresponding volume of RBCs to keep the cell concentration in the probe constant. An increase in the SLI corresponded to cell volume increase, SLI decrease corresponded to hemolysis; **(d)** – The SLI at each osmolality step was analyzed according to 1.2 section, and the corresponding cell volume distributions were calculated using LaSca software v.1498.

1.2 Determination of the absolute RBC volume by laser diffraction

For the scattered light intensity conversion in absolute mean RBC volume (MCV), we calibrated the laser particle analyzer using the carboxyl-modified latex beads of 3, 6, 10, and 16 μm size. The light scattering indicatrix was analyzed at angles from 0.75 to 12° separately for each bead type (Supplementary Figure S2A) according to [11]. Then, each indicatrix was linearized and compared to the linearized indicatrices of RBCs and platelets (Supplementary Figure S2B). These results indicated that according to the cell size and

scattering indicatrix, the angles in the range from 0.75 to 4° are sensitive to cell volume changes for the objects with 3-8 μm size. The scattering angles ranging from 6 to 8° were insensitive to cell volume changes and, therefore could characterize cell rupture more accurately. As RBCs and platelets are nonspherical particles, then calibration curve according to the scattered light intensities corresponding to the particle sizes might give inaccurate results. Therefore, here we calibrated the diameters according to the corresponding carboxyl beads ratio of indicatrices which is more complex and accurate. Using this approach, we depicted the analyzed particles according to their diameters (Supplementary Figure S2C). The mean cell volumes and their distribution were then recalculated from the obtained diameters by the LaSca software (Figure 2B, C).

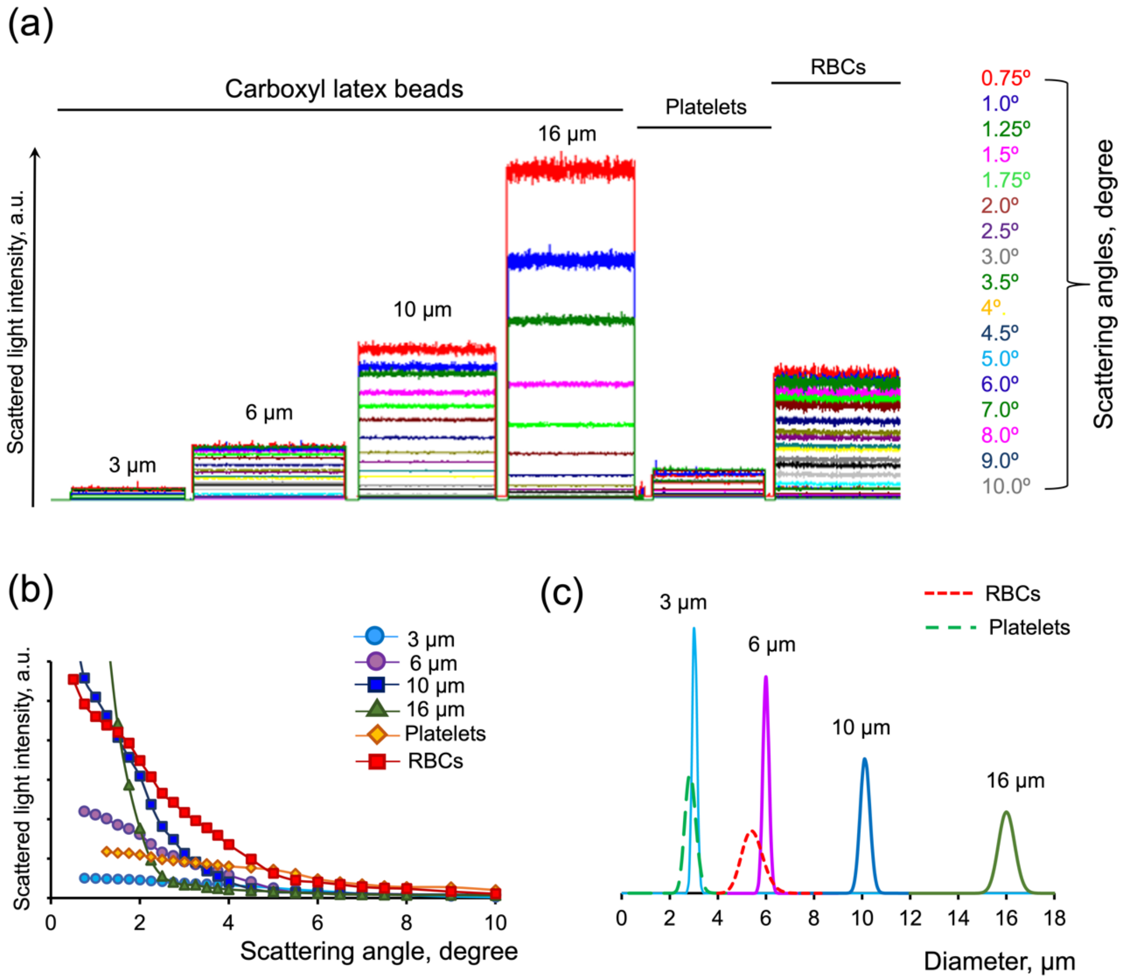

Supplementary Figure S2. Development of the approach for absolute cell volume estimation. (a) – carboxyl latex beads, washed platelets (10^6 cells/mL), or washed RBCs (10^6 cells/mL) were suspended as indicated in HEPES buffer, and the corresponding scattered light intensity was registered; (**b**) – the scattered light intensities from S2A were plotted against the scattering angles to obtain the corresponding scattering indicatrix; (**c**) – in accordance with the ratio of indicatrices for the analyzed carboxyl beads, RBCs and platelets were plotted on a scale for the diameters**.**

According to the data from Sections 1.1 and 1.2, we developed an approach for absolute cell volume determination using the laser diffraction method. Hereinafter, in the AST model, we used flow cytometry for hemolysis detection according to the distribution of the events in the FSC/SSC coordinates and laser diffraction for the characterization of the AM transport kinetics and absolute cell size estimation.

1.3 Estimation of absolute cell volume changes and determination of kinetic parameters for AM-triggered cell swelling and hemolysis quantitative analysis

To develop an algorithm for quantitative characterization of AM-triggered cell swelling and hemolysis, we analyzed the kinetics of the AST reaction and found that (i) before the hemolysis, the dynamics of SLI changes at 2.5° angle (Supplementary Figure S3c) corresponded to the changes in cell volume

(Supplementary Figure S3b-d), (ii) SLI changes at 6.5-7° angles were insensitive to cell swelling although sensitive to the hemolysis. Based on these observations, we established the kinetic parameters to characterize AM-triggered cell swelling and hemolysis (Supplementary Figure S3d-e): V_{Sw} – the initial rate of cell swelling, V_{hem} – the maximal rate of hemolysis, and % Hemolysis – the percent of hemolyzed cells. To calculate the instant rates from the hemolysis curve, we differentiated it to obtain the values for the corresponding extrema (Figure 2e).

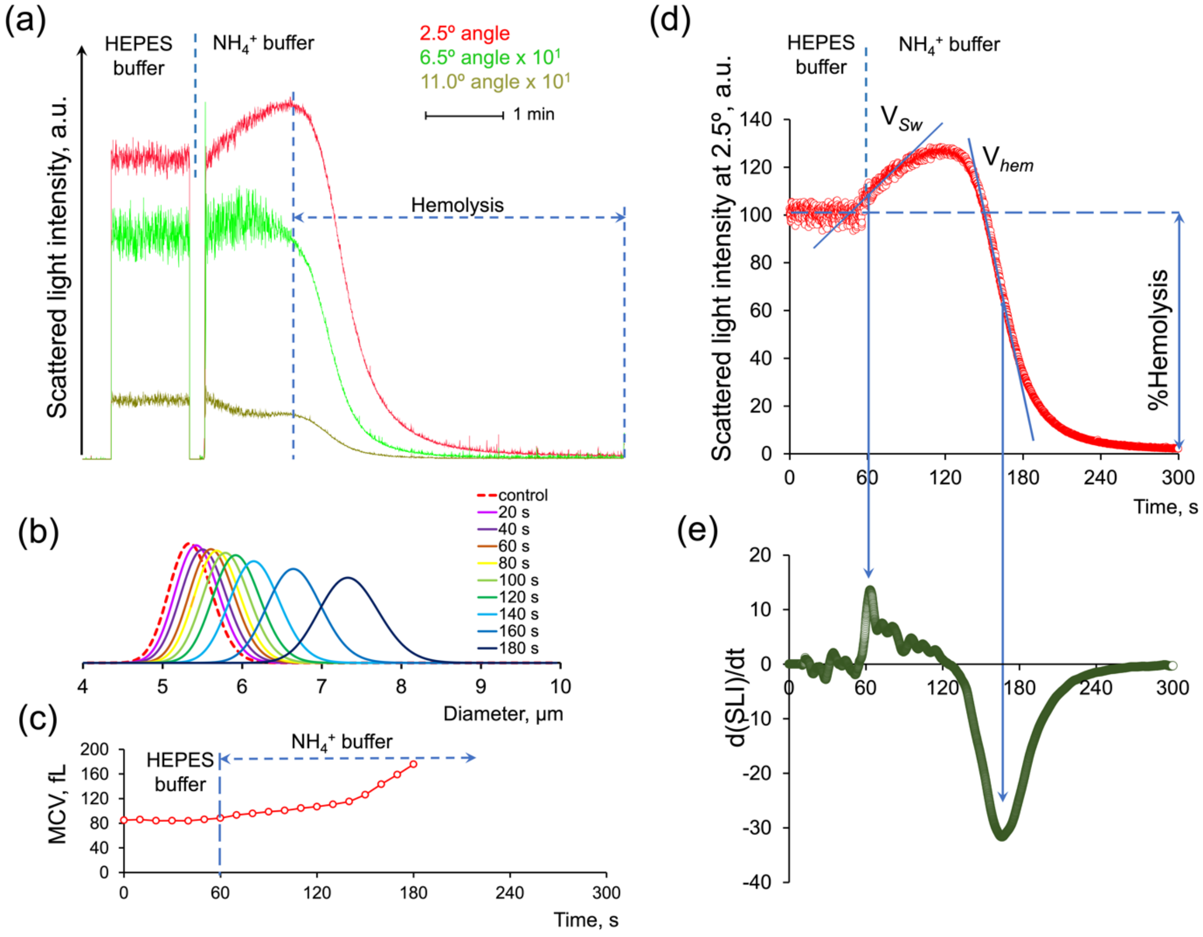

Supplementary Figure S3. Quantitative characterization of NH_4^+ - induced cell swelling and hemolysis using the laser diffraction technique. (a) – washed RBCs (10^6 cells/mL) were suspended in HEPES or NH_4^+ buffer as indicated and analyzed by laser diffraction method. Scattered light intensity (SLI) from RBCs suspended in HEPES buffer at 2.5, 6.5, and 11° angles were registered for 2 min for control. Then, the RBCs (10^6 cells/mL) were suspended in isotonic NH_4^+ buffer, and SLI registration continued for 4 min, SLI increase at 2.5° angle corresponded to cell swelling, SLI decrease throughout the scattering angles – to hemolysis. (**b**) – distribution of RBCs by diameters every 20 s during AST; (**c**) – mean corpuscular volume of RBCs (MCV) in kinetics calculated by the LaSca algorithm from Fig. 1B according to CML beads calibration curve; (**d**) – the original record of SLI changes (hemolysis curve) at 2.5° registered by the LaSca-TM laser particle analyzer. Graphic explanation of the quantification of AST parameters by original software of the LaSca analyzer (LaSca v.1498) from the hemolysis curves: $\mathbf{V}_{Sw}$ – initial rate of cell swelling; $\mathbf{V}_{hem}$ – maximal rate of hemolysis; **%Hemolysis** - % of hemolyzed cells; (**e**) – Differentiation of hemolysis curve for determination of extrema: maximum, corresponding to V_{Sw}, and minimum, corresponding to V_{hem}.

Therefore, here we developed the original approach for absolute cell volume determination using the laser diffraction method along with flow cytometry.

2 Chloride anion substitution has no negative effects on RBC viability and Hb state.

To test whether the concentration of another primary eAE1 ligand, chloride anion, alters AM-induced cell swelling and hemolysis, we substituted chloride ions with glutamate ions (see Methods 4.1, MSG, and MAG buffers) and initially analyzed whether chloride-free conditions affect cell viability or hemoglobin (Hb) oxidation state during the time of our experiments. Cell viability in chloride-free buffers was investigated using cell esterase activity, and Hb forms were registered spectrophotometrically. Preincubation of RBCs with tert-butyl hydroperoxide (*t-BOOH*) induces oxidative stress, unstable Hb species formation, and decreases intracellular esterase activity [10, 12], therefore, damaged by oxidative stress RBCs were used as a negative control.

First, using hypotonic hemolysis and hemolysis in NH_4^+ buffer to generate cell-free Hb we showed that AST has no negative effects on Hb (Supplementary Figure S4a). Next, we showed that such markers of metHb formation as the increase in absorption in the red region 600-650 nm or the flattening of the region of characteristic peaks of oxy-Hb (541 and 577 nm), were not detected in RBCs suspended in MSG or MAG buffers either (Supplementary Figure S4b). In chloride-free buffers, the spectra of Hb encapsulated within RBC were shifted vertically upward (Supplementary Figure S4b, lines 3 and 4), indicating that the RBCs most likely underwent spherization in the absence of Cl^-, i.e., under conditions of inverted chloride gradient. Using flow cytometry and fluorometry, we showed that compared to *t-BOOH*-treated RBCs, neither MSG buffer nor MAG buffer caused a decrease in calcein fluorescence intensity, indicating that intracellular esterase activity was not depleted (Supplementary Figure S4c, d).

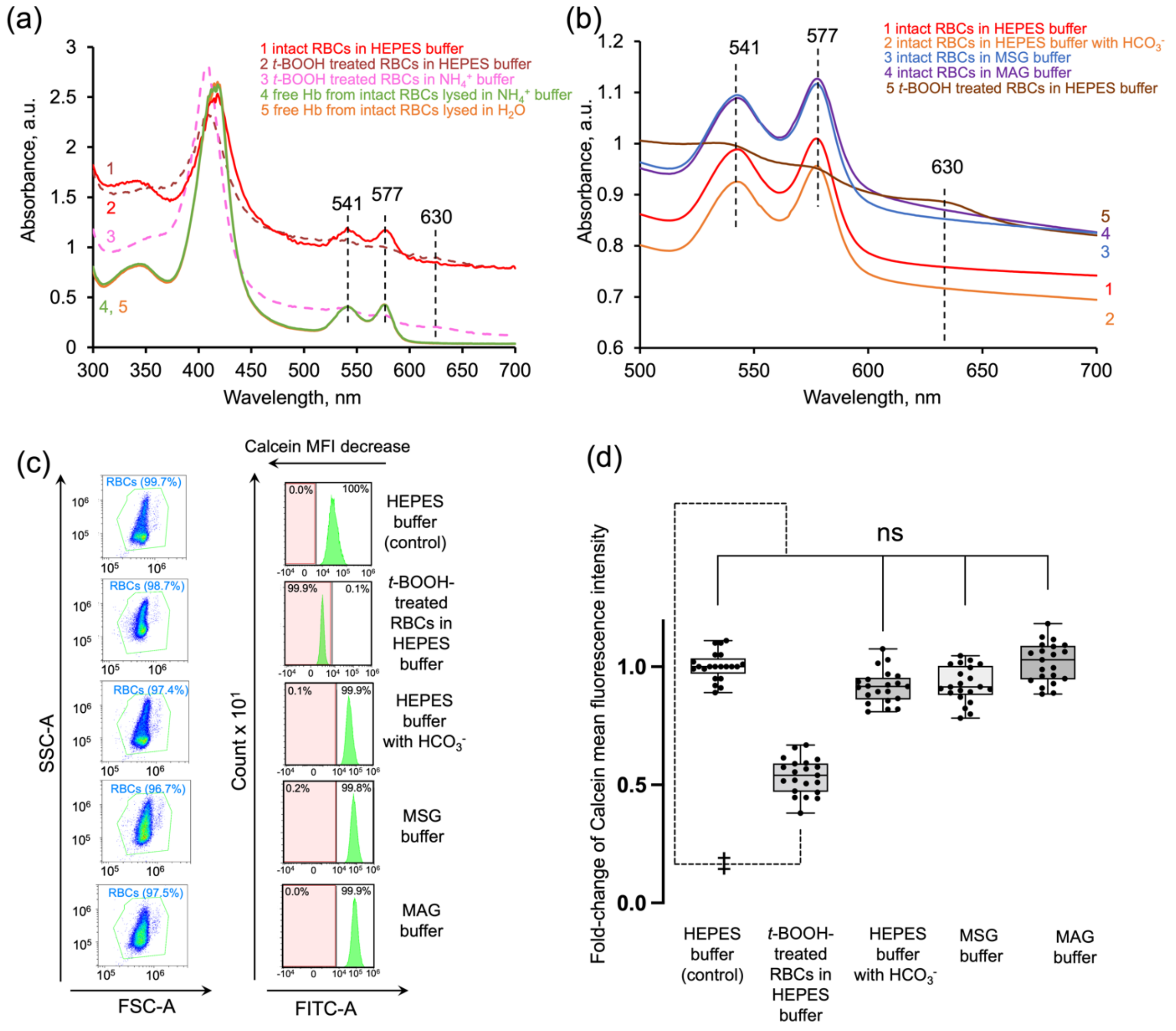

Supplementary Figure S4. Chloride ion substitution with glutamate ions does not induce the formation of unstable Hb or decrease RBC esterase activity. **(a)** - Washed RBCs (10^6 cells/mL) or *t-BOOH*-treated RBCs (final concentration 1.5 mM, 60 min at 37°C) were suspended in HEPES buffer, NH_4^+ buffer, or dH_2O, and placed in a 96-well plate with the following scanning for spectra of cell-encapsulated Hb from 300 to 700 nm in HEPES buffer and cell-free Hb in NH_4^+ buffer or dH_2O; **(b)** - Intact RBCs (10^6 cells/mL) and RBCs treated with *t-BOOH* (10^6 cells/mL) were suspended in

HEPES, HEPES with HCO_3^-, MSG and MAG buffers during 15 min, placed in 96-well plate and scanned for spectra of cell-encapsulated Hb in the range from 300 to 700 nm (spectra of one representative experiment from 10). **(c, d)** - Washed RBCs (10^6 cells/mL) incubated with calcein-AM (5 μM, 30 min, 37°C) were suspended in HEPES buffer, HEPES buffer with HCO_3^-, MSG, and MAG buffers. For the negative control, RBCs dyed with calcein-AM and treated with *t*-BOOH (1 mM, 1 h) were suspended in HEPES buffer 10^6 cells/mL. Then, the calcein fluorescence intensity was analyzed by (A) flow cytometry (CytoFLEX flow cytometer) or (B) fluorescence spectroscopy (CLARIOstar microplate reader, BMG). (**c**) – pseudo-color plots and histograms of a representative experiment of 5; **(d)** – C-AM-stained intact or *t*-BOOH-treated RBCs were placed in a 96-well plate in indicated buffers and were analyzed fluorometrically. Data are presented as Mean ± SD, n=9, One way ANOVA with Tukey *post hoc*, ns, not significant, ‡, $p<0.001$;

Our data indicated that both MSG and MAG buffers do not decrease RBC viability or induce unstable Hb formation and, therefore, are appropriate for Cl^- substitution experiments.

3 Calculation of intracellular AM concentration

To calculate the estimated intracellular AM concentration, which corresponds to the experimentally observed fall in NH_4^+ concentration in the supernatant (Figure 9), we composed the balanced equation according to the ratio of extracellular (V_{out}) to intracellular (V_{in}) water and $[NH_4^+]$ changes in the extracellular space:

$$[NH_4^+]_{in} = [NH_4^+]_{in}^0 + \frac{V_{out}}{V_{in}}([NH_4^+]_0 - [NH_4^+]_{out}),$$ where

$[NH_4^+]_{in}$ – the current intracellular ammonium concentration; parameter of our interest;
$[NH_4^+]_{out}$ – the current ammonium concentration in media; experimentally detected;
V_{out}/V_{in} – the ratio of extracellular to intracellular water;
$[NH_4^+]_{in}^0$ – the initial intracellular ammonium concentration;
$[NH_4^+]_0$ –NH_4Cl 800 μM; which was added to the media.

We used RBCs in concentration 1.2 × 10^9 cells/mL in the experiment. Initial MCV, according to the hematological analyzer, was 84 fL. Therefore, the calculated hematocrit is 10% HCT. Then, the ratio of V_{out} to V_{in} is estimated as V_{out}/V_{in} = 9:1.

If postulate that intracellular and extracellular concentrations were equal, assuming extracellular $[NH_4^+]$ within physiologically relevant plasma range (below 60 μM) [13], e.g., 30 μM, then the initial conditions are as follows:

$[NH_4^+]_{in}^0$ = 30 μM, $[NH_4^+]_0$ = 30 μM

Then we washed the RBCs, got rid of extracellular AM, and resuspended cells, assuming that there is no immediate AM efflux:

$[NH_4^+]_{in}^0$ = 30 μM, $[NH_4^+]_0$ = 0 μM

Next, we added 800 μM NH_4Cl:

$[NH_4^+]_{in}^0$ = 30 μM, $[NH_4^+]_0$ = 800 μM

Then, according to the balanced equation, the dynamics of intracellular ammonium concentration was estimated quantitatively (Supplement Figure S5).

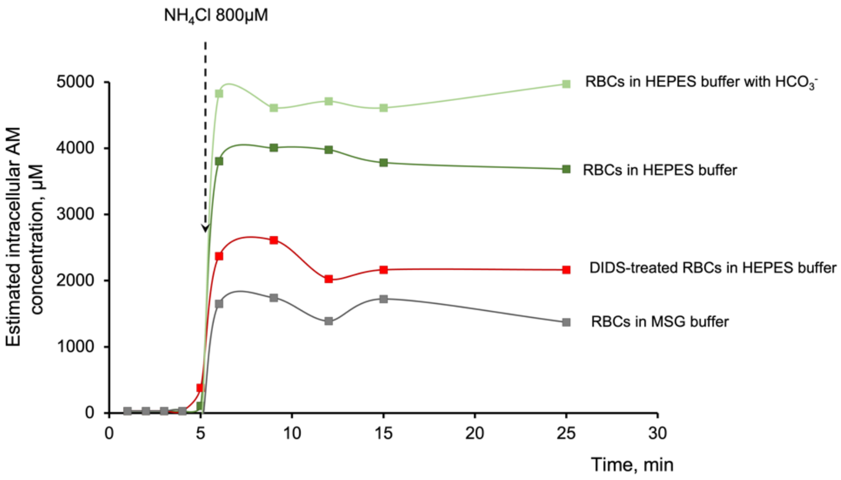

Supplementary Figure S5. RBCs are able to accumulate AM from the media in case of exceeding the physiological AM range. Washed RBCs (1.2 x 10^9 cells/mL) were suspended in HEPES buffer (violet squares), HEPES buffer with DIDS (red circles), HEPES buffer with HCO_3^- (blue squares), and MSG buffer (grey circles) at 37°C. 4 aliquots of each probe were collected at 1, 2, 3, 4 min time points, immediately centrifuged, and the supernatant was collected for further AM detection. Then NH_4Cl (800 μM) was added to the samples, and aliquots were taken in (in min): 0.5, 1, 5, 10, 15, and 25 - after addition of AM, immediately centrifuged at 4°C with a supernatant collection for further AM concentration assay. The data were then processed through the balanced equation to calculate the corresponding to such NH_4^+ decrease in supernatant increase in intracellular NH_4^+ concentration. Data are presented as Mean ± SD, n=6. One way ANOVA for multiple comparisons, Leven's test $p<0.05$, Tamhane T2 *post hoc*, *, $p<0.05$ compared to preliminary controls (the first four points).

Therefore, experimentally determined decrease of ammonium concentration in supernatant could lead to intracellular ammonium concentration increase up to at least: 3.7 ± 0.4 mM in HEPES buffer (intact RBCs), 4.9 ± 0.6 mM in HEPES buffer with HCO_3^- (intact RBCs), 2.2 ± 0.2 mM in HEPES buffer (RBCs pre-incubated with DIDS), 1.4 ± 0.2 in MSG buffer (intact RBCs) (n=5, $p<0.05$). However, if consider the dry matter of the cell and basic high intracellular [NH_4^+], these values would be even greater.

4 Carbonic anhydrase II inhibition leads to reduced cell swelling and hemolysis velocity, however, does not block it completely

Carbonic anhydrase II (CAII) activity was shown to be tightly connected to the eAE1 function; therefore, this enzyme is most likely indirectly involved in AM transport in human RBCs. To check this hypothesis, we inhibited CAII with acetazolamide and performed the AST. The rates of cell swelling and hemolysis were significantly reduced in RBCs treated with AAZ compared to the control RBCs.

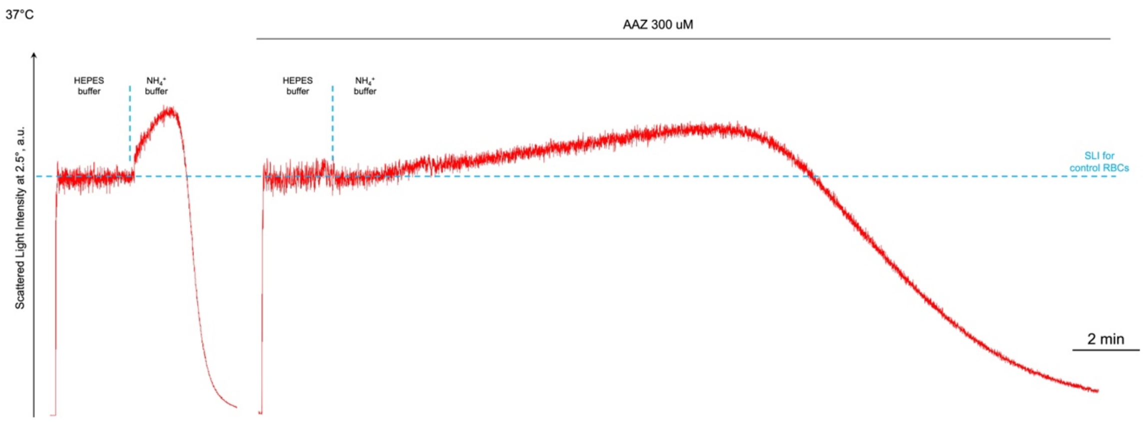

Supplementary Figure S6. Inhibition of CAII by acetazolamide reduces the velocity of cell swelling and hemolysis in AST. Washed RBCs (10^6 cells/mL) or acetazolamide (AAZ) - treated RBCs (final concentration 300 uM, 10 min, 37°C) were suspended in HEPES buffer, or NH_4^+ buffer, as indicated. Representative experiment, one of 6;

These data indicate that CAII is also involved in AM transport in human RBCs, however the mechanisms are not clear yet and merit future examinations.

SUPPORTING REFERENCES

1. Gienger, J.; Gross, H.; Ost, V.; Bar, M.; Neukammer, J., Assessment of deformation of human red blood cells in flow cytometry: measurement and simulation of bimodal forward scatter distributions. *Biomed Opt Express* **2019,** 10, (9), 4531-4550.
2. Tzur, A.; Moore, J. K.; Jorgensen, P.; Shapiro, H. M.; Kirschner, M. W., Optimizing optical flow cytometry for cell volume-based sorting and analysis. *PLoS One* **2011,** 6, (1), e16053.

3. Friebel, M.; Helfmann, J.; Meinke, M. C., Influence of osmolarity on the optical properties of human erythrocytes. *J Biomed Opt* **2010,** 15, (5), 055005.
4. van der Pol, E.; van Leeuwen, T. G.; Yan, X., Misinterpretation of solid sphere equivalent refractive index measurements and smallest detectable diameters of extracellular vesicles by flow cytometry. *Sci Rep* **2021,** 11, (1), 24151.
5. Brittain, G. C. t.; Chen, Y. Q.; Martinez, E.; Tang, V. A.; Renner, T. M.; Langlois, M. A.; Gulnik, S., A Novel Semiconductor-Based Flow Cytometer with Enhanced Light-Scatter Sensitivity for the Analysis of Biological Nanoparticles. *Sci Rep* **2019,** 9, (1), 16039.
6. Roma, P. M.; Siman, L.; Hissa, B.; Agero, U.; Braga, E. M.; Mesquita, O. N., Profiling of individual human red blood cells under osmotic stress using defocusing microscopy. *J Biomed Opt* **2016,** 21, (9), 90505.
7. Reinhart, W. H.; Piety, N. Z.; Goede, J. S.; Shevkoplyas, S. S., Effect of osmolality on erythrocyte rheology and perfusion of an artificial microvascular network. *Microvasc Res* **2015,** 98, 102-7.
8. Pribush, A.; Meyerstein, D.; Meyerstein, N., Kinetics of erythrocyte swelling and membrane hole formation in hypotonic media. *Biochim Biophys Acta* **2002,** 1558, (2), 119-32.
9. Mindukshev, I.; Kudryavtsev, I.; Serebriakova, M.; Trulioff, A.; Gambaryan, S.; Sudnitsyna, J.; Khmelevskoy, D.; Voitenko, N.; Avdonin, P.; Jenkins, R.; Goncharov, N., Flow cytometry and light scattering technique in evaluation of nutraceuticals. In *Nutraceuticals: Efficacy, Safety and Toxicity*, 2016; pp 319-332.
10. Sudnitsyna, J.; Skverchinskaya, E.; Dobrylko, I.; Nikitina, E.; Gambaryan, S.; Mindukshev, I., Microvesicle Formation Induced by Oxidative Stress in Human Erythrocytes. *Antioxidants (Basel)* **2020,** 9, (10).
11. Mindukshev, I. V.; Krivoshlyk, V. V.; Ermolaeva, E. E.; Dobrylko, I. A.; Senchenkov, E. V.; Goncharov, N. V.; Jenkins, R. O.; Krivchenko, A. I., Necrotic and apoptotic volume changes of red blood cells investigated by low-angle light scattering technique. *Spectroscopy* **2007,** 21, 105-120.
12. Welbourn, E. M.; Wilson, M. T.; Yusof, A.; Metodiev, M. V.; Cooper, C. E., The mechanism of formation, structure and physiological relevance of covalent hemoglobin attachment to the erythrocyte membrane. *Free Radic Biol Med* **2017,** 103, 95-106.
13. Barsotti, R. J., Measurement of ammonia in blood. *The Journal of Pediatrics* **2001,** 138, (1), S11-S20.
